# Peripheral autoreactive CD8 T‐cell frequencies are too variable to be a reliable predictor of disease progression of human type 1 diabetes

**DOI:** 10.1002/cti2.1309

**Published:** 2021-07-11

**Authors:** Johnna D Wesley, Susanne Pfeiffer, Darius Schneider, David Friedrich, Nikole Perdue, Birgit Sehested‐Hansen, William Hagopian, Matthias G von Herrath

**Affiliations:** ^1^ Novo Nordisk Research Center Seattle, Inc. Seattle WA USA; ^2^ La Jolla Institute La Jolla CA USA; ^3^ Pacific Northwest Research Institute Seattle WA USA; ^4^ Novo Nordisk A/S Søborg Denmark

**Keywords:** autoimmunity, biomarker, peripheral immune cell composition, prediction, type 1 diabetes

## Abstract

**Objectives:**

The detection of a peripheral immune cell signature that specifically reflects autoimmunity in type 1 diabetes would enable the prediction and staging of disease on an individual basis. However, defining such a signature is technically challenging. Reliable interpretation of immune cell‐related biomarkers depends on their inherent variability and, to understand this variability, longitudinal analyses are required.

**Methods:**

We performed a longitudinal observational study in which 40 individuals with elevated genetic risk of type 1 diabetes and persistent islet autoantibodies provided a blood sample every 4–6 weeks for > 1 year.

**Results:**

Peripheral immune cell composition (T cells, NK cells and monocytes) was assessed using well‐validated flow cytometry panels and demonstrated that, while non‐antigen‐specific immune cell subsets were stable over time, autoantigen‐reactive T‐cell frequencies were highly variable in and between individuals. Neither the frequency nor phenotype of non‐antigen‐specific subsets or autoreactive CD8^+^ T cells associated with clinical onset of T1D.

**Conclusion:**

The findings from the **T**ype **1 D**iabetes Longitudinal **BI**omarker **T**rial underscore the inherent challenge of evaluating changes in peripheral immune cell populations as surrogates of organ‐specific disease activity. The variability of peripheral antigen‐specific T cells precludes their use as a prognostic marker and clearly demonstrates that a reliable prognostic cell signature remains elusive.

## Graphical abstract

We searched for a peripheral immune cell signature that predicts the onset of type 1 diabetes and performed a longitudinal study in individuals at risk of developing type 1 diabetes. Non‐antigen‐specific immune cell subsets were stable and autoantigen‐reactive T‐cell frequencies were highly variable over time.

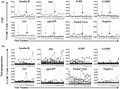

## Introduction

Type 1 diabetes (T1D) is characterised by autoimmune‐mediated destruction of pancreatic beta‐cells and loss of glucose‐stimulated insulin responses leading to chronic hyperglycaemia.^1^, ^2^ A variety of immune cells infiltrate the islets of Langerhans during disease development. These infiltrates have been extensively studied *in vivo* and *ex vivo*. In the non‐obese diabetic (NOD) mouse, a commonly used model of T1D, CD4^+^ T cells comprises the majority of the islet infiltrate early in disease development and CD8^+^ T cells mediate beta‐cell death, demonstrating a clear T‐cell‐dependent mechanism in this model.^3^, ^4^ In humans, these infiltrates are dominated by autoreactive CD8^+^ T cells^5^, ^6^, ^7^ that are considered to be the principal mediators of beta‐cell dysfunction and death.^8^, ^9^

Although T cells are the main drivers of disease progression, the main peripheral biomarkers used for marking T1D progression are islet autoantibodies. Unfortunately, autoantibodies change relatively slowly and are poorly informative regarding specific timing of disease onset in individuals. Reliable T‐cell‐related biomarkers would be of great use in disease progression monitoring, refining disease risk or especially predicting individual timing of onset. The peripheral frequency of islet‐specific T cells in humans is very low, and similar autoreactive peripheral T cells may be found in healthy individuals without diabetes, making the interpretation of their direct role in the disease difficult^10^. Hence, it has not been possible to pinpoint a specific peripheral immune cell signature in subjects at risk of T1D. Earlier studies evaluating causal relationships between disease progression and peripheral immune cell signature have been cross‐sectional, with few or no longitudinal samples from the same individual.^11^, ^12^, ^13^ Longitudinal evaluations would be far more relevant than single timepoint sampling to detect changes in peripheral immune cell frequencies and phenotypes associated with disease mechanisms, prognosis and timing of progression. Therefore, we conducted the first longitudinal study to specifically evaluate the inherent variation of peripheral immune cell subsets (autoreactive CD8^+^ T cells, broad T‐cell memory, NK and monocyte cell subsets) in prediabetic individuals as part of the **T**ype **1 D**iabetes Longitudinal **Bi**omarker **T**rial (T1DBIT). We evaluated immune subsets that have been previously studied in cross‐sectional assessments of immune status in humans at risk of developing T1D or who had been diagnosed with T1D.^11^, ^12^ Samples from individuals at high risk of developing T1D were collected monthly over the course of 1 year, or, if less frequently, up to 2 years. In six cases, this included samples before, at and after diagnosis of T1D. The assays used in this study included a high level of quality control as a necessary prerequisite to determine biological variability. The aim of this study was to evaluate the variation in peripheral immune profiles that could indicate progression to T1D. The data generated from this study were intended to be used as a benchmark in future immune intervention trials to allow for more reliable monitoring of immune deviation, better patient selection, improved overall assessment of efficacy and a more reliable interpretation of T cell‐related biomarkers. While multiple studies have been conducted to understand immune changes in the periphery, to our knowledge, this is the first‐time longitudinal sampling and in‐depth profiling of immune phenotype was performed in individuals at high risk of progression to T1D.

## Results

Demographics of the 40 analysed subjects are provided in Table 1. The mean age of these 40 participants was 14.7 years at enrolment (range: 4.3–36.6 years), with 22 females and 18 males. During the study period, five males and one female were diagnosed with T1D. Note that only 10 of 40 subjects had a first‐degree relative (FDR) with T1D, including 2 of 6 subjects who were diagnosed with T1D during the study period. Information regarding the participant's HLA and insulin promoter VNTR is shown in Supplementary table 1. Individual HbA1c values at all timepoints are shown in Supplementary figure 1.

**Table 1:** Participant demographics

	All		Type 1 diabetes^a^		Non‐progressors^a^ ^,^ ^b^	
	*N*	%	*N*	%	*N*	%
Number of subjects	40	100	6	100	34	100
Male + female	18 + 22	43 + 57	5 + 1	83 + 17	13 + 21	36 + 64
Ethnicity						
Hispanic or Latino	2	10	0	0	2	10
Not Hispanic or Latino	34	81	6	100	28	78
Not known	4	10	0	0	4	11
Subject with a first‐degree relative with T1D	10	24	2	33	8	22
HLA‐A*02:01	23	58	2	33	21	62

	Mean	SD	Mean	SD	Mean	SD
Age (years)	14.7	6.6	14.3	2.6	15.4	7.3
Height (m)	1.58	0.20	1.66	0.15	1.57	0.21
Weight (kg)	56.01	19.69	58.29	16.96	55.61	20.32

Islet autoantibodies present at screening	*n*	%	*n*	%	*n*	%
GADA + IA2A + IAA + ZnT8A	1	3	1	17	0	0
GADA + IA2A + IAA + ZnT8A	5	13	2	33	3	9
GADA + IA2A + ZnT8A	10	25	3	50	7	21
GADA + IAA + ZnT8A	2	5	0	0	2	6
GADA + IAA	2	5	0	0	2	6
GADA + IA2A	1	3	0	0	1	3
GADA + ZnT8A	2	5	0	0	2	6
IA2A + ZnT8A	3	8	1	17	2	6
IAA + ZnT8A	1	3	0	0	1	3
GADA only	3	8	0	0	3	9
IA2A only	1	3	0	0	1	3
IAA only	10	25	0	0	10	29

^a^ Type 1 diabetes refers to enrolled individuals who were diagnosed during the study period; Non‐progressors refers to participants who remained at risk but did not progress to type 1 diabetes during the study period.

^b^ Two subjects withdrew consent because of personal reasons on days 304 and 87, respectively.

At the time of enrolment, subjects were initially screened for the presence of autoantibodies to GAD65 (GADA), insulin (IAA), insulinoma antigen 2 (IA2A) and zinc transporter 8 (ZnT8A; Table 1). Notably, all subjects who developed T1D during the study were positive for multiple islet autoantibodies at enrolment (Table 1). Further, all subjects underwent islet autoantibody measurement at every follow‐up visit and remained autoantibody‐positive for one or more antibodies throughout the entire observation period (data not shown).

### Broad populations of T cells and innate immune cells remained stable during the study

**Figure 1:**
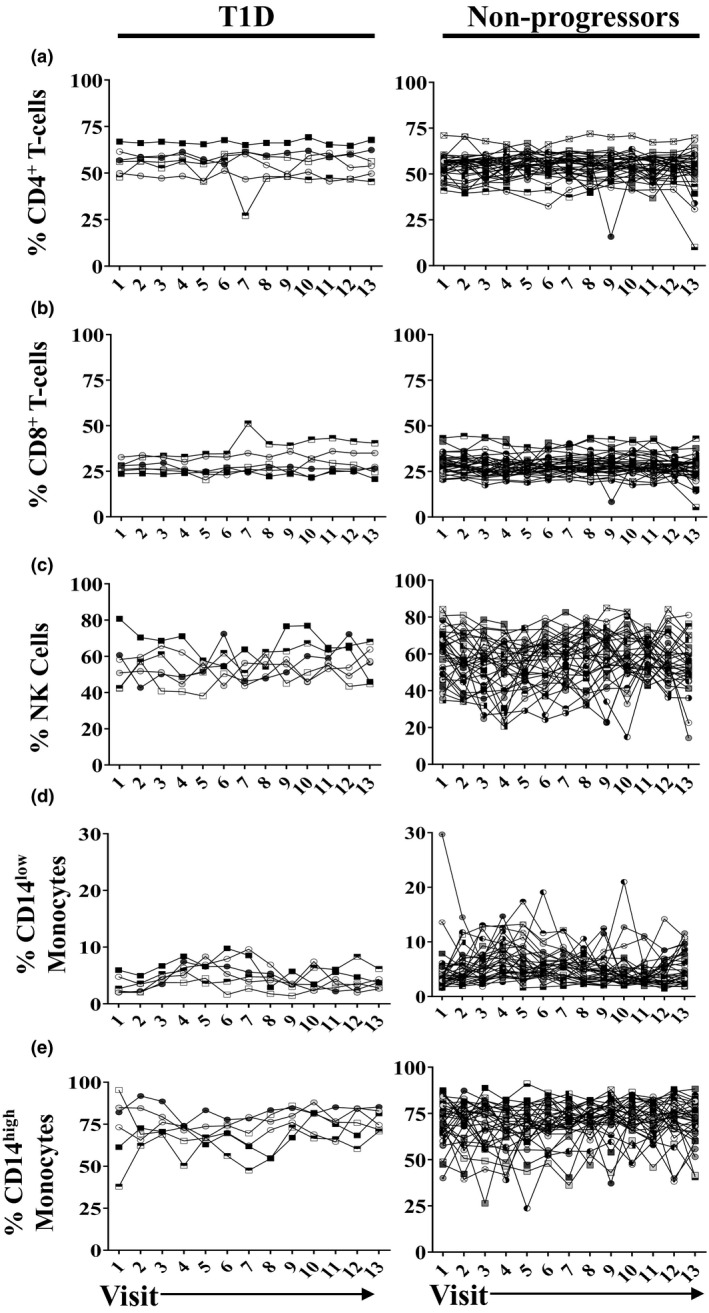
The frequency of total circulating immune subsets remained fairly constant overtime within an individual, regardless of diabetes status. All analysed study participants are shown; each line represents an individual. The frequency of total **(a)** CD4^+^CD8^−^ T cells; **(b)** CD4^−^CD8^+^ T cells; **(c)** NK cells; **(d)** CD14^low^ monocytes; and **(e)** CD14^hi^ monocytes is shown. Type 1 diabetes refers to enrolled individuals who were diagnosed during the study period; Non‐progressors refers to participants who remained at risk of type 1 diabetes and did not progress.

**Figure 2:**
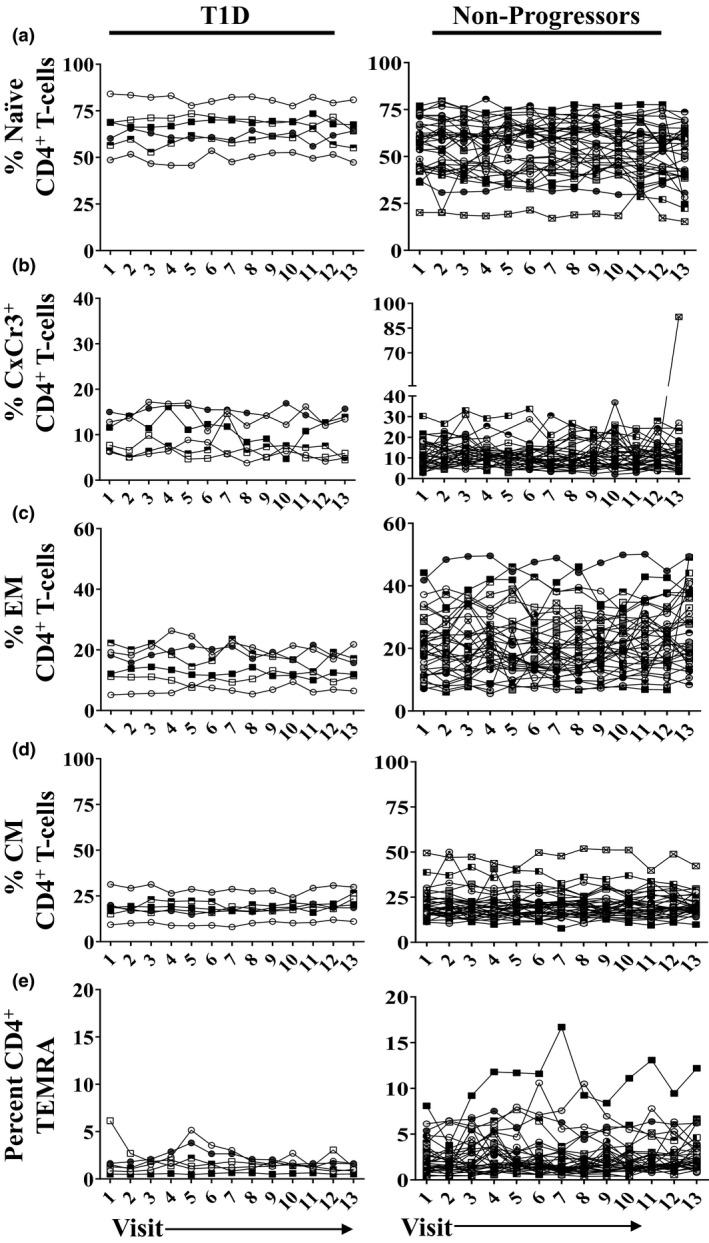
The frequency of total circulating CD4^+^ T‐cell subsets is stable over time in an individual. Each line represents a single participant. The frequency of total **(a)** naive CD4^+^ T cells; **(b)** CxCr3^+^ CD4^+^ T cells; **(c)** effector memory CD4^+^ T cells; **(d)** central memory CD4^+^ T cells; and **(e)** terminally differentiated CD45RA^+^ effector memory (TEMRA) CD4^+^ T cells is shown over time. Subjects who were diagnosed with T1D during the study (left panel, T1D; *n* = 6) and subjects who remained at risk and did not progress (right panel, non‐progressor; *n* = 34) are shown.

**Figure 3:**
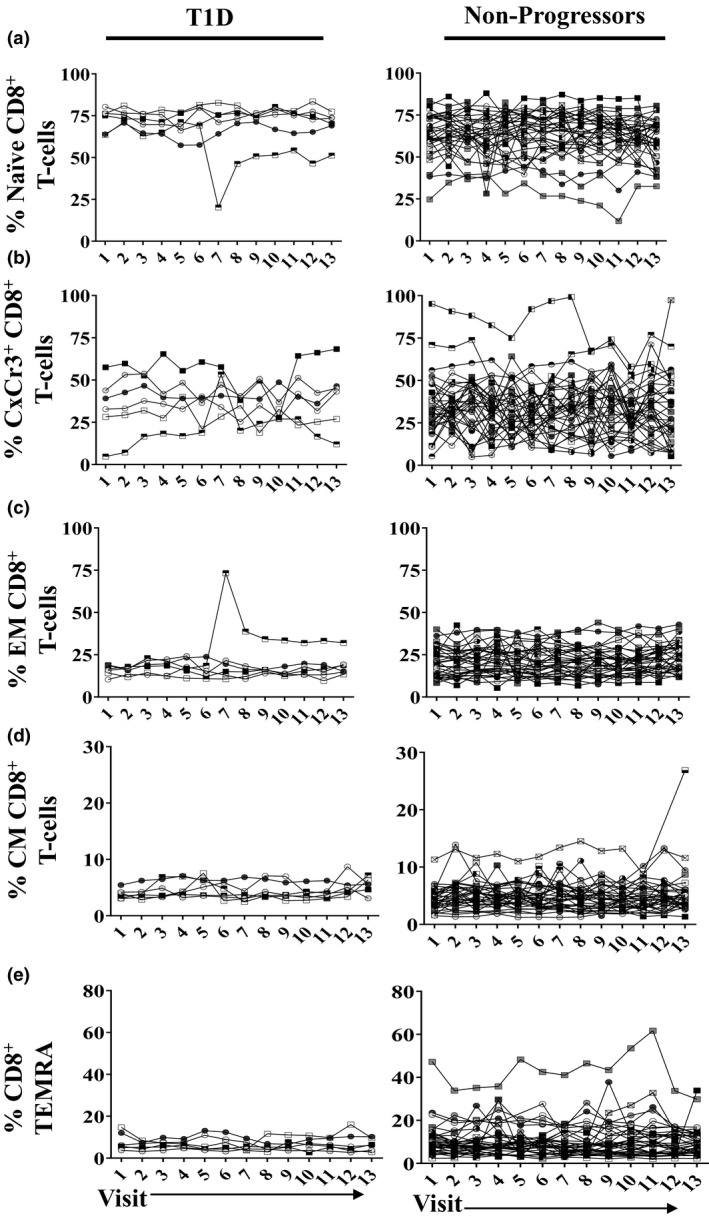
The frequency of total circulating CD8^+^ T‐cell subsets is stable over time in an individual. Each line represents a single participant. The frequency of total **(a)** naive CD8^+^ T cells; **(b)** CxCr3^+^ CD8^+^ T cells; **(c)** effector memory CD8^+^ T cells; **(d)** central memory CD8^+^ T cells; and **(e)** terminally differentiated CD45RA^+^ effector memory CD8^+^ T cells are shown over time. Subjects who were diagnosed with T1D during the study (left panel, T1D; *n* = 6) and subjects who remained at risk and did not progress (right panel, non‐progressor; *n* = 34) are shown.

In general, the frequency of global T‐cell populations varied little within an individual over the year of study (Figures 1, 2, 3; Supplementary figures 2 and 3), regardless of disease diagnosis. The associated CV was low, as expected, for the general populations [i.e. naïve, T_H_1 activated + CXCr3^+^, effector memory; central memory; and terminal effector memory (TEMRA)]. The populations with the highest CV were also those with the lowest peripheral frequency (Table 2; Figures 2, 3). CD4^+^ and CD8^+^ naïve T cells were associated with lower median CV values in T1D^+^ group compared to subjects that were not diagnosed with T1D during the study (non‐progressors, NP group), although this may be related to small sample size. Additionally, CXCr3^+^ CD8^+^ T cells were also less variable in T1D^+^ group compared to those who were not diagnosed during the study period (Table 2), and circulating NK cell and monocyte populations were similar between the groups in both frequency and CV (Figure 1, Table 2; Supplementary figures 2–4). During our observational study, six of 40 individuals developed T1D. We were unable to establish any correlation between any immune cell subset and disease onset. No major changes within these peripheral immune cell subsets were observed that would aid in the prediction of the timing of disease onset (Supplementary figures 2–4).

**Table 2:** Median %CV of peripheral immune subsets across all visits

Population	Type 1 diabetes^a^			Non‐type 1 diabetes^a^		
	*n*	Median %CV	Interquartile range	*n*	Median %CV	Interquartile range
Live cells	6	7.9	7.3; 8.9	34	10.6	7.8; 14.9
Total CD4^+^CD8^−^ T cells	6	5.1	3.7; 6.9	34	5.8	4.0; 9.3
CD4^+^ naïve T cells	6	3.8	2.8; 5.5	34	7.3	5.4; 11.6
CD4^+^ central memory T cells	6	10.2	7.1; 11.3	34	11.3	9.2; 13.5
CD4^+^ CXCr3^+^ T cells	6	24.6	15.4; 25.1	34	**28.4**	21.8; 38.0
CD4^+^ effector memory T cells	6	13.4	10.8; 16.7	34	16	12.7; 23.4
CD4^+^ TEMRA	6	**30.9**	25.0; 51.5	34	**40.2**	29.2; 46.9
Total CD4^−^CD8^+^ T cells	6	6.1	5.5; 11.0	34	8.9	7.0; 10.8
CD8^+^ naïve T cells	6	4.9	3.9; 6.8	34	8.4	6.7; 11.5
CD8^+^ central memory T cells	6	**25.6**	10.2; 34.0	34	**25.9**	19.5; 33.3
CD8^+^ CXCR3^+^ T cells	6	17.7	12.8; 20.8	34	**29.6**	21.0; 36.5
CD8^+^ effector memory T cells	6	14.2	13.5; 18.6	34	15.4	13.9; 18.5
CD8^+^ TEMRA	6	22.8	19.5; 29.5	34	23.5	18.8; 31.8
IA2^+^ CD8^+^ T cells	2	**70.3**	63.0; 90.2	21	**81.9**	59.6; 100.4
PPI^+^ CD8^+^ T cells	2	**81.8**	27.7; 95.5	21	**77.1**	66.0; 104.9
ppIAPP^+^ CD8^+^ T cells	2	**106.3**	86.9; 116.9	21	**99.1**	67.3; 131.5
InsB^+^ CD8^+^ T cells	2	**94.5**	63.1; 120.7	21	**85.4**	60.2; 97.0
IGRP^+^ CD8^+^ T cells	2	**145.9**	99.9; 265.4	21	**116.5**	67.9; 139.8
GAD65^+^ CD8^+^ T‐ cells	2	**90.5**	69.5; 98.7	21	**87.1**	73.5; 114.9
NK cells	6	14.2	12.1; 15.6	34	16.3	12.5; 20.6
CD2^+^ NK cells	6	4.1	2.6; 6.5	34	2.8	1.9; 4.9
CD36^+^ NK cells	6	23.1	19.5; 33.5	34	**27.1**	22.3; 31.5
CD54^+^ NK cells	6	8.5	2.5; 13.8	34	4.8	2.1; 8.2
CD57^+^ NK cells	6	6.9	3.6; 12.3	34	4.8	3.5; 6.8
NKG2D^+^ NK cells	6	**35.7**	29.1; 41.2	34	**37.5**	31.0; 52.6
NKp46^+ ^NK cells	6	3.9	1.7; 11.5	34	3.1	0.8; 6.7
CD56^hi^ NK cells (NK^hi^ cells)	6	21.5	18.4; 24.0	34	**25.3**	20.2; 28.4
CD2^+^ NK^hi^ cells	6	1.2	0.9; 1.3	34	0.7	0.5; 1.2
CD36^+^ NK^hi^ cells	6	**39.5**	34.4; 42.7	34	**36.2**	30.8; 44.4
CD54^+^ NK^hi^ cells	6	1.1	0.2; 2.2	34	0.6	0.3; 0.9
CD57^+ ^NK^hi^ cells	6	**56.1**	49.1; 76.1	34	**69.8**	56.2; 83.8
NKG2D^+^ NK^hi^ cells	6	7.7	5.9; 13.2	34	7.3	4.9; 13.1
NKp46^+^ NK^hi^ cells	6	2.4	1.9; 4.6	34	1.9	1.0; 2.8
CD14^hi^ monocytes	6	9.8	6.4; 11.3	34	11.1	8.7; 14.9
CD2^+^ CD14^hi^ monocytes	6	**42.5**	37.0; 67.0	34	**47.1**	38.0; 57.3
CD36^+^ CD14^hi^ Monocytes	6	0.7	0.2; 2.8	34	0.6	0.2; 0.9
CD57^+^ CD14^hi^ Monocytes	6	**53.3**	49.3; 60.2	34	**47.9**	38.3; 67.3
HLA Class II^+^ CD14^hi^ monocytes	6	12.2	10.0; 14.3	34	13.9	10.2; 17.8
PDL1^+^ CD14^hi^ monocytes	6	19.3	12.5; 21.6	34	18.3	14.8; 22.0
CD14^low^ monocytes	6	**36.3**	34.5; 45.0	34	**41.2**	31.2; 51.4
CD2^+^ CD14^low^ monocytes	6	**89.5**	57.8; 151.5	34	**73.8**	61.1; 111.0
CD36^+^ CD14^low^ monocytes	6	5.5	3.7; 12.1	34	5.1	3.9; 7.1
CD57^+ ^CD14^low^ monocytes	6	**61.1**	57.6; 66.4	34	**50.1**	44.6; 62.9
HLA Class II^+^ CD14^low^ monocytes	6	11.6	10.0; 18.1	34	12.9	9.9; 17.3
PDL1^+^ CD14^low^ monocytes	6	17.1	14.7; 22.5	34	19.6	16.4; 27.0

Broad population analyses were performed for all 40 subjects who completed the study. For the antigen‐specific CD8^+^ T‐cell analyses (IA2^+^ CD8^+^ T cells; PPI^+^ CD8^+^ T cells; ppIAPP^+^ CD8^+^ T cells; InsB^+^ CD8^+^ T cells; IGRP (islet‐specific glucose‐6‐phosphatase)^+^ CD8^+^ T cells; GAD65^+^ CD8^+^ T cells, only samples from the 23 participants who were HLA‐A2:01^+^ were analysed. Subject‐specific CVs across all visits were summarised across subjects using median values and interquartile ranges as shown in the table.

^a^ Type 1 diabetes refers to enrolled individuals who were diagnosed during the study period; Non‐progressors refers to participants who remained at risk but did not progress to type 1 diabetes during the study period. Bold text indicates %CV ≥ 25.

### Circulating islet autoreactive CD8^+^ T cells were infrequent and highly variable over time

**Figure 4:**
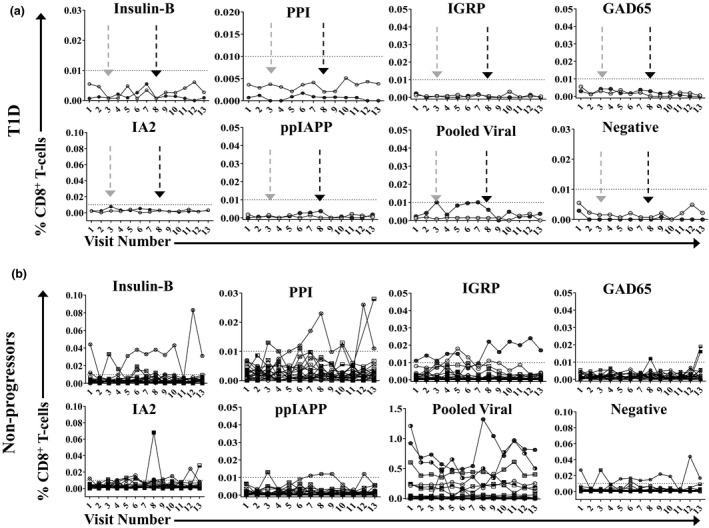
Autoantigen‐specific circulating CD8^+^ T‐cell subsets are not present in most subjects, and when detected are highly variable. All HLA*A2:01^+^ subjects are shown. Each line represents a single participant. The frequency of autoreactive CD8^+^ T cells in the HLA*A2:01^+^ subset is shown for those who were diagnosed with T1D during the study (left panel, T1D; *n* = 2) and for the subjects who remained at risk and did not progress (right panel, non‐progressor; *n* = 21). The grey and black arrows indicate the first visit post‐diagnosis for each subject, respectively. The dotted line indicates lower limit of detection (0.01%) for multimer assay. Note that pooled viral multimers included multimers for CMV pp65, EBV LMP2 and measles H250.

The majority of HLA‐A2:01^+^ study participants (*n* = 23; including 2 T1D^+^ subjects) were negative for all autoreactive populations evaluated. Samples from neither of the HLA‐A2:01^+^ participants diagnosed with T1D during the study (*n* = 2) contained more than 0.01% of non‐naïve multimer^+^ CD8^+^ T‐cell populations at any point during the study when evaluating the individual multimer + populations. Twelve at‐risk subjects (out of the 21 HLA‐A2:01^+^ non‐progressors) were positive for an autoreactive CD8^+^ T‐cell population with a frequency of > 0.01% at one or more visits and were most commonly positive for InsB‐ or PPI‐specific CD8^+^ T cells (Figure 4, Table 2, Supplementary table 2). While none of those 12 subjects were positive at all 13 visits, five subjects had at least one autoreactive CD8^+^ T‐cell population detected at ≥ 3 visits. Pooling all positive events for the six autoreactive multimers provided a cumulative frequency ranging from 0.002% to 0.019% for the T1D^+^ subjects and a range of 0.00157–0.1506% for the non‐progressors (Supplementary figures 5 and 6). Comparing the autoreactive CD8^+^ T‐cell frequency between non‐progressors who were positive for single autoantibody at enrolment to those who were positive for multiple autoantibodies did not demonstrate any significant differences (Supplementary figure 7).

Unfortunately, the CV for all autoreactive T‐cell subsets examined was exceedingly high across the longitudinal sampling, likely because of the rarity of the populations evaluated (Table 2; Figure 4). Surprisingly, GAD65‐reactive CD8^+^ T cells were the most infrequent population detected—only three participants tested positive and only at a single visit (Figure 4; Supplementary table 2). Because of the high variability and limited positive events, no correlation between circulating autoantigen T‐cell frequency and any clinical parameter was detectable.

## Discussion

Islet autoantibodies are widely accepted as a defining characteristic of T1D and are almost always present before onset.^2^ However, a peripheral T‐cell signature or biomarker that reflects islet autoimmunity flare or remission could allow for much better timing of disease activity and progression at the individual level, with greater precision than autoantibodies since the latter are slow to change over time. Better prognostic timing could limit severe complications at diagnosis and contribute to a better understanding of the pathologic process of disease. Additionally, a reliable prognostic biomarker may aid in patient selection for improved statistical power in immunotherapy trials,^14^ aid in better selection of immunotherapeutic agents and aid in optimising dosage and schedule of administration. This biomarker would need to be responsive to the progression and therapeutic modulation of autoimmunity and be sufficiently stable to be interpretable within and between individuals or populations in low sample volumes with limited sampling frequency.

Circulating biomarkers evolve over time, like the underlying pathology they are intended to reflect, and the inherent variation of such biomarkers is key to their utility in prognosis or prediction. Therefore, we performed longitudinal sampling and analyses with stringent quality checks, assay controls and well‐validated assay methods with low inter‐ and intra‐assay variability to evaluate the variation of key immune cell subsets implicated in islet autoimmunity that held promise as predictive and prognostic biomarkers.^15^, ^16^, ^17^ We found that the broad, non‐antigen‐specific CD4^+^ and CD8^+^ T‐cell subsets and overall innate immune subsets had remarkably low variation within individuals. The greatest variation was observed in low‐frequency populations such as terminally differentiated CD4^+^ CD45RA^+^ memory cells (40%) and senescent innate subsets: CD57^+^ CD56^hi^ NK cells, CD57^+^ CD14^low^ and CD57^+^ CD14^hi^ monocytes (66.9%, 49.5% and 53.4% CV, respectively, among all participants and visits, Table 2and data not shown). This variation was consistent regardless of T1D status, and none of these populations appeared to associate with T1D diagnosis or clinical parameters of diabetes in the current analyses, keeping in mind that this study was not powered to evaluate these associations.

Published data to date suggest that the peripheral T‐cell repertoire is not fully shared with the pancreas or lymphoid tissue, nor is the peripheral frequency of subsets similar to solid tissue.^18^ For example, antigen‐specific memory T cells in peripheral blood comprise only, at most, 2.5% of circulating T cells; in contrast, memory cells comprise the majority of T cells found in solid tissues.^19^ Additionally, autoantigen‐specific CD8^+^ T cells can be found in the peripheral blood of healthy individuals.^20^ However, we had hoped that the autoantigen‐specific CD8^+^ T‐cell frequency would provide a hint of disease activity and impending disease in the pre‐onset period when islet destruction was presumably most active and that the inherent variation in a circulating autoreactive population would not preclude its usefulness in disease prediction. We evaluated the most commonly studied autoantigen‐reactive CD8^+^ T cells (HLA‐A2:01‐restricted) using flow cytometry and a well‐validated and controlled multimer assay that was intended for clinical trial use. Overall, the autoantigen‐specific T‐cell frequency was sporadic and plagued with excessive variation. Although repeated responses to the same antigen were detectable in seven out 21 HLA‐A2:01^+^ subjects, these recurring responses showed an intermittent pattern over time in the positive individuals. Few subjects were positive for peripheral GAD65‐ or ppIAPP (islet amyloid polypeptide)‐reactive CD8^+^ T cells. Reactivity to IA2, PPI and insulin B, while more common, was still significantly variable and poorly informative. It should be noted that studies using the NOD mouse have also highlighted the sporadic nature of autoantigen‐specific T cells.^21^ The longitudinal changes seen in rodents may mirror what is seen in humans.

Although autoreactive CD8^+^ T cells were detected in individuals who did not progress to T1D during our study, as others have shown in non‐risk healthy individuals, most of those who progressed to disease were negative for all autoreactive T‐cell subsets measured both before and after onset.^22^ This could mean that the immune process in T1D is fully sequestered within the pancreas and associated lymph nodes, and little of the relevant specific cellular autoimmunity is detectable in peripheral blood. If disease‐driving cells exist in peripheral circulation, they are rare and current cytometry technology and methods are not sufficient to specifically detect them. Alternatively, our findings may suggest that peripheral antigen‐specific T cells that are also found in healthy individuals do not contribute to disease progression. This brings into question the usefulness of measuring these cells as a predictive marker in clinical trials using current methods, even using a carefully designed, validated and controlled version of the antigen‐specific multimer assay widely reported in T1D studies.^23^, ^24^Previously published studies, using different methods, have provided insight regarding the possibility of a prognostic circulating biomarker.^25^, ^26^, ^27^, ^28^ Most of these studies have focused on sequencing of fractionated and/or *in vitro* stimulated cells, reporting gene signatures linked to longitudinal progression or single timepoint‐based prediction. While these published reports provided some positive findings regarding the possibility of a composite biomarker, they also reported that there are few differences between individuals with T1D, those at risk and those who are healthy with no predefined susceptibility, similar to our current findings. The main differences between our study and published biomarker studies evaluating T1D progression are the method of autoreactive T‐cell evaluation, post‐isolation stimulation of the T cells and frequency of sampling. Our broader approach of analysing the immune cell frequencies without sorting or *in vitro* stimulation of T cells may have limited our ability to detect or amplify rare signals. However, at the time, we were more concerned with potentially detecting false‐positive signals that might have arisen with *ex vivo* stimulation and focused on characterising the immune subsets in a way that would be more broadly applicable to a larger clinical trial setting.

There are two major limitations to the current study: key phenotypic and functional markers were not included in the original cytometric analyses and the length of the observation period. This study was analysed in 2015, and we did not include markers related to senescence and exhaustion that have been identified in the intervening years. The original focus of this study was to evaluate non‐regulatory, antigen‐specific T cells and innate immune cells. Our primary interest at the time of the study was to characterise the variation in the frequency of autoreactive T‐cell populations and innate cell subsets. We did not evaluate intracellular markers during the initial analyses due to our primary interest in autoreactive T‐cell frequency and the need to prioritise samples for these analyses. Additional evaluation using the remaining cryopreserved samples could be considered in the future. Furthermore, because of the length of the study observation period, only six subjects of 40 developed clinical T1D and only two were positive for HLA‐A2:01, limiting our evaluation of autoreactive CD8^+^ T cells.

However, a major strength is the longitudinal sampling—including 13 timepoints per individual, with samples preceding, at and after diagnosis for the subjects diagnosed with diabetes—and the validation associated with our methods and data generation. Single timepoint analysis in this context may have resulted in a different interpretation of the results depending on the timepoint analysed (Figure 4; Supplementary figures 2–7; Supplementary table 2). Cross‐sectional studies, especially those evaluating antigen‐specific T cells, at a single timepoint per individual may not accurately reveal to true composition of peripheral immune cells or their relationship to autoimmune disease development.^11^, ^12^, ^13^ The current approach allowed for in‐depth immune phenotype profiling per individual over time and aided in our evaluation of inherent immune stability, variation and timing of these signals as compared to disease progression.

This study highlights the challenge of attempting to evaluate organ‐specific disease mechanisms using peripheral samples and underscores the need for using well‐controlled assays in a longitudinal set‐up to address these technically challenging questions. As technology and methods continue to advance, there may be more reliable, clinically feasible methods for identifying peripheral biomarkers that strongly correlate with progression or point the way to targetable pathways that control the elusive initiating event for T1D.

Collectively, the current findings remind us that much remains to be learned about the precise mechanisms of beta‐cell loss, the role of various immune populations in islet autoimmunity and how to harmlessly monitor intrapancreatic processes during the development and implementation of immunotherapy to delay or prevent clinical T1D. Additional longitudinal analyses, sharing of best practices for assay development and implementation and data sharing between industry and academic researchers are required to bridge the gap in our understanding to achieve clinical success.

## Methods

### Study participants

The T1DBIT Study (NCT01846312) prospectively enrolled 42 subjects who were positive for ≥ 1 islet autoantibody [among GADA (anti‐GAD65 autoantibody), IA2A (anti‐insulinoma antigen 2 autoantibody) and/or IAA (anti‐insulin autoantibody)] from 2013 to 2015 at a single centre, the Pacific Northwest Research Institute (PNRI) in Seattle, Washington, USA. Subjects had previously taken part in either the Diabetes Evaluation in Washington (DEW‐IT) Study or the Washington State Diabetes Prediction Study.^29^, ^30^ Eligible subjects were volunteers in general good health, ≥ 4 years or < 40 years of age, weighed ≥ 16.7 kg (37 pounds) and were positive (> 99th percentile) for ≥ 1 of 3 islet autoantibodies (IAA, GADA or IA2A). We excluded any individual who had a disease that might jeopardise their safety or protocol compliance; females who were pregnant or breastfeeding; and any subject taking a medication that could influence the immune system within the 4 weeks prior to enrolment, including steroids, methotrexate, sulfasalazine, hydroxychloroquine, cyclosporine, azathioprine or penicillamine.

The study was designed to follow and frequently sample, over a 12‐ to 18‐month period, high‐risk individuals who were not yet diagnosed with diabetes at the time of enrolment. Participants who developed T1D during the study period continued the same sampling schedule through at least 13 total visits. Enrolled subjects were studied for up to 1.5 years from enrolment at 4‐ to 6‐week intervals. Study participants provided up to 25 mL of blood samples at each follow‐up visit at PNRI, the participant’s home or off‐site at a clinical laboratory licensed for phlebotomies. Forty‐two subjects were enrolled, and two subjects withdrew during the study; therefore, 40 participants completed all visits and were included in study analyses. There were 22 females and 18 males enrolled in the study. The study was conducted under continuous Institutional Review Board approval, with voluntary informed consent by all adult participants or, for children, by their parent or legal guardian. Children ≥ 7 years of age were also required to sign a voluntary assent form prior to participation. Participant demographics are shown in Table 1.

### Whole‐blood processing

Prior to cell isolation, heparinised blood samples were centrifuged at 600 × *g* to gently separate plasma. After removal of < 50% of the plasma volume, sterile PBS (GIBCO, Thermo Fisher Scientific, Waltham, Massachusetts, USA), equivalent in volume to the amount of plasma removed, was added to the plasma‐depleted blood and inverted to mix. Plasma was stored at −80 °C until needed. Blood samples from a single individual were then pooled in a single tube, further diluted 1:1 with sterile PBS and mixed gently. Up to 30 mL of diluted blood was then pipetted onto Ficoll‐Paque Plus (GE Healthcare, Chicago, Illinois, USA) in LeucoSep tubes (Greiner, Kremsmünster, Austria). Tubes were centrifuged at 1000 × *g* for 12 min at room temperature. The plasma‐enriched upper layer was removed to approximately 5 mm above the cell layer. The cell layer was removed and washed in excess PBS. After centrifugation at 250 × *g*, supernatant was decanted and the cell pellet was gently resuspended in phenol red‐free RPMI 1640 media supplemented with 10 mL 1 M HEPES (GIBCO/Thermo Fisher Scientific, Waltham, Massachusetts, USA). Cells were counted using standard methods, pelleted and gently resuspended in human AB serum (Life Technologies, Carlsbad, California, USA) at a cell concentration of ≥ 20 × 10^6^ cells mL^−1^ for cryopreservation. An equal amount of human AB serum (Life Technologies, Carlsbad, California, USA) supplemented with 80% cell culture‐grade DMSO (Sigma‐Aldrich, St. Louis, Missouri, USA) was added dropwise to the cell suspension. The suspension was aliquoted into labelled 1.8‐mL cryovials; each cryovial contained at least 1 mL (10 × 10^6^ cells). Cryovials were transferred to a cryopreservation container (Biocision, Brooks Lifesciences, Chelmsford, Massachusetts, USA) as per the manufacturer’s instructions to ensure even freezing at −80 °C. Vials were transferred to liquid nitrogen within 5 days for long‐term storage prior to analyses.

### Autoantibody detection

Autoantibodies to human islet autoantigens GAD65, IA2, ZnT8 and insulin were measured using an analogous procedure.^31^ Briefly, GAD651‐585, IA2608‐979 and ZnT8 JH6.2 hinged dimer were separately biosynthetically labelled with ^35^S‐methionine (Amersham Pharmacia Biotech) by coupled in vitro transcription and translation (TNT, Promega, Madison, Wisconsin, USA) using expression plasmids derived from cDNAs cloned from human islets.^32^, ^33^, ^34^ ^125^I‐monoiodo‐TyrA_14_‐human insulin was purchased from Perkin‐Elmer (Waltham, Massachusetts, USA). Antigens were diluted in assay buffer (PBS/Tween or Tris/Tween) and used in separate assays at 20,000 to 50,000 cpm in triplicate wells. Sera were added at 2 µL, 2 µL, 5 µL and 2.5 µL per well for GAD65, IA2, insulin and ZnT8, respectively. After mixing, antigen and serum were incubated at 4 °C for 24‐72 h to form immune complexes that were then captured and counted in 96‐well multiscreen filter membrane microtiter plates coated with protein A (EMD Millipore, Burlington, Massachusetts, USA). Plates were gently mixed for 30 min at 4 °C on an orbital plate shaker (Perkin‐Elmer, Waltham, Massachusetts, USA), washed 12 times by vacuum aspiration and counted on a Microbeta counter (Perkin‐Elmer, Waltham, Massachusetts, USA) as detailed elsewhere.^31^ Evaluation in the IASP 2018 Proficiency Testing showed performance specificities of 100%, 98.8%, 100% and 97.8% for GAD65, IA2, insulin and ZnT8, respectively.

### Healthy control samples for assay development and implementation

For all flow cytometry assays, anonymous whole‐blood samples, up to 100 mL, were purchased from Astarte Biologics (Bothell, Washington, USA), Research Blood Components (Boston, Massachusetts, USA) or BloodWorksNW (Seattle, Washington, USA) and processed for cryopreservation at the Novo Nordisk Research Center Seattle, Inc. (NNRCSI) as described above for the study samples. These samples were used for assay development and as assay run controls during study analyses.

### Thawing cryopreserved PBMC (peripheral blood mononuclear cells)

Cryopreserved PBMC tubes were rapidly thawed at 37 °C in a water bath; the tube then sprayed with 70% ethanol to sterilise and transferred to a biosafety cabinet. PBMCs were resuspended in 9‐mL warm complete media (phenol red‐free 1640 RPMI (GIBCO/Thermo Fisher Scientific, Waltham, Massachusetts, USA) supplemented with 2% GlutaMAX, 2% HEPES and 10% human AB serum (all from Life Technologies) containing 15 U Benzonase (Novagen, Madison, Wisconsin, USA) per mL cell suspension in a 15‐mL conical tube. PBMC samples were centrifuged for 10 min at 300 × *g,* and the supernatant was discarded. For flow cytometry assays, the PBMCs were resuspended in 1 mL of warm complete medium and immediately evaluated for cell concentration and viability using the Guava ViaCount Assay (Millipore, Burlington, Massachusetts, USA). Cells resuspended at 10 × 10^6^ cells mL^−1^ in 100 μL (NK/Monocyte panel) or in 200 μL (QDM panel) aliquots were transferred to separate wells of a 96‐well round‐bottom plate.

### NK/monocyte flow cytometry (NK/Mono)

After thawing, 1 × 10^6^ PBMC was incubated with LIVE/DEAD Aqua Dead cell stain (Molecular Probes, Eugene, Orlando, USA) for 30 min at room temperature. Fluorochrome‐conjugated antibodies were then added for 30 min at room temperature; all antibodies are listed in Supplementary table 3. Data were acquired on an LSRII (BD Biosciences, Franklin Lakes, New Jersey, USA) and analysed using FlowJo software (BD, Ashland, Oregon, USA). The gating schema and representative flow cytometry data are shown in Supplementary figure 8; the parameter name and gating details are given in Supplementary table 4.

### Qdot‐multimer flow cytometry (QDM)

Antigen‐specific CD8^+^ T‐cells were evaluated using a modified version of a previously described method.^14^, ^32^ Peptide‐HLA‐A02:01 (pHLA) complexes were generated by Novo Nordisk as described in Hadrup *et al*.^14^ All monomers were stored at −80 °C until multimerised. Complexes were multimerised and labelled with quantum dots in a 96‐well plate kept at 4 °C using a 96‐well cold block (Biocision, Brooks Lifesciences, Chelmsford, Massachusetts, USA). Prior to multimerisation, the concentration of each monomer was confirmed using a Nanodrop (Thermo Scientific, Waltham, Massachusetts, USA). All streptavidin‐conjugated quantum dots (Qdots, Life Technologies, Carlsbad, California, USA) were stored at 4 °C and were centrifuged at 13,000 rpm (15,871 × *g*) for 1 min at room temperature with high break to pellet aggregates prior to use. Multimer specificity and sensitivity were tested prior to study using primary T‐cell clones spiked into a negative donor PBMC and, when possible, previously identified positive and negative donors. After thawing, 1 μL protease of inhibitor cocktail set I (EMD Millipore, Burlington, Massachusetts, USA) was added to 10 μg monomers. Streptavidin‐conjugated Qdot reagents (Life Technologies, Carlsbad, California, USA) were diluted 1:5, added to the monomer/protease inhibitor and then mixed gently at 4 °C for 20 min. This procedure was repeated four more times for a total of five 20‐min incubations of the step‐wise addition of streptavidin‐conjugated Qdots. Then, D‐biotin (Avidity, Aurora, Colorado, USA) was added at a final concentration of 80 μM and cultured with agitation at 4 °C for 20 min. The multimers were aliquoted in single‐use aliquots and frozen in PBS at −80 °C for up to 6 months before use. Qdot combinations per pHLA are shown in Supplementary table 5.

After PBMCs were thawed and counted, the cell suspension volume was adjusted to 10 × 10^6^ cells mL^−1^ phenol red‐free1640 RPMI (GIBCO/Thermo Fisher Scientific, Waltham, Massachusetts, USA) supplemented with 2% GlutaMAX (Life Technologies, Carlsbad, California, USA), 2% HEPES (GIBCO/Thermo Fisher Scientific, Waltham, Massachusetts, USA) and 10% human AB serum (Life Technologies, Carlsbad, California, USA). Then, 200 μL of cell suspension was added per well (2 × 10^6^ cells per well) to a sterile 96‐well round‐bottom plate. The plate was centrifuged for 2 min at 300 × *g* with high break at room temperature. The media was removed and cells were resuspended in either LIVE/DEAD Aqua (Molecular Probes) or PBS (GIBCO/Thermo Fisher Scientific, Waltham, Massachusetts, USA) and incubated in the dark for 30 min at room temperature. PBS was added to the wells and the plate was centrifuged twice. After the second centrifugation, cells were incubated with 500 nM dasatinib (LC Laboratories, Woburn, Massachusetts, USA) for 30 min at 37 °C. The cells were centrifuged for 2 min at 300 × *g* with high break at room temperature. Prepared multimers were centrifuged for 1 min at 13,000 × *g* at room temperature to remove aggregates before incubation with cells. Multimers were added to the wells and incubated with the cells for 10 min at 37 °C; fluorescence minus one (FMO) controls for all multimers were incubated with FACS buffer for 10 min at 37 °C instead of multimers. Cells were then incubated in PBS supplemented with 2% human AB serum (Life Technologies, Carlsbad, California, USA) and additional fluorochrome‐conjugated antibodies for 30‐min at room temperature; all antibodies and cytometer filter set‐up are listed in Supplementary table 6. Data were acquired on an LSRII (BD Biosciences, Franklin Lakes, New Jersey, USA) and analysed using FlowJo software (BD, Ashland, Oregon, USA). For analyses, dead cells were excluded using the LIVE/DEAD stain. Doublet discrimination was accomplished using forward scatter height versus area dot plots, and monocyte, NK‐cell, B‐cell and dendritic cell populations were excluded from the analyses using FITC‐conjugated anti‐CD14, anti‐CD16, anti‐CD56, anti‐CD20, and anti‐CD40. Fluorescence‐minus‐one (FMO) controls were used to set all gates during analyses. Data were acquired on an LSRII (BD Biosciences, Franklin Lakes, New Jersey, USA) and analysed using FlowJo software (BD, Ashland, Oregon, USA). For analyses, dead cells were excluded using the LIVE/DEAD stain. Fluorescence‐minus‐one (FMO) controls were used to set all gates during analyses. The gating schema and representative flow cytometry data are shown in Supplementary figures 9 and 10; the parameter name is provided in Supplementary table 7.

### LSRII QC procedure

All experiments for this study were performed on two five‐laser 20‐parameter LSRII flow cytometers with matching configurations. CST beads were run daily on both machines. Additionally, Ultra Rainbow Fluorescent Particles‐Mid Range (Spherotech, Lake Forest, Illinois, USA) were used daily to QC individual parameters. MFI target values for these beads were previously determined for each parameter. Application‐specific optimisation of PMT voltages was performed to ensure electronic noise minimally contributed to detected signal as recommended by the manufacturer. PMT voltages were adjusted daily to match predetermined MFI target values.

### Flow cytometry data collection & analyses

Data were acquired on an LSRII (BD Biosciences, Franklin Lakes, New Jersey, USA) and analysed using FlowJo software (BD, Ashland, Oregon, USA). For analyses, dead cells were excluded using the Live/Dead stain. Doublet discrimination was accomplished using forward scatter height versus area dot plots. For the NK/Mono panel, B‐cell and T‐cell populations were excluded from the analyses using fluorochrome‐conjugated anti‐CD19 and anti‐CD3, respectively; and for the QDM, monocyte, NK‐cell, B‐cell and dendritic cell populations were excluded from the analyses using FITC‐conjugated anti‐CD14, anti‐CD16, anti‐CD56, anti‐CD20 and anti‐CD40. For both panels, 1 × 10^6^ events were acquired for the samples, and 5 × 10^5^ events were acquired for each FMO. FMO controls were used to set all gates during analyses.

### Genotyping of participants

HLA‐DRB1, DQA1 and DQB1 genotyping utilised direct sequencing of exon 2 of each gene to identify specific alleles. After study enrolment, subjects were typed at the insulin promoter type 1 diabetes risk locus using a −21 Hph RFLP method,^36^ and additional low‐resolution typing was done on HLA Class I genes A, B and C. Later, high‐resolution genotyping of HLA‐A2^+^ study participants was done by BloodWorksNW (Seattle, Washington, USA) to identify individuals carrying the HLA‐A02:01:01 allele necessary for QDM analyses. Results are shown in Supplementary table 1.

### Statistical analyses

To quantify temporal intra‐individual variation of cellular subsets, subject‐specific coefficients of variation (CV) were calculated as the average cellular level divided by the standard deviation across visits. Subject‐specific CVs were then summarised across subjects using median values and interquartile ranges. Note that all individuals completing visit 13 were included in the analyses and defined as ‘completers’.

## Conflict of Interest

The authors declare no conflict of interest.

## Author Contributions

**Johnna D Wesley:** Conceptualization; Data curation; Formal analysis; Funding acquisition; Investigation; Methodology; Project administration; Resources; Software; Supervision; Validation; Visualization; Writing‐original draft; Writing‐review & editing. **Susanne Pfeiffer:** Writing‐original draft; Writing‐review & editing. **Darius Schneider:** Data curation; Formal analysis; Software; Visualization. **David Friedrich:** Data curation; Formal analysis; Software; Validation; Visualization. **Nikole Perdue:** Data curation; Formal analysis; Investigation; Methodology; Writing‐original draft. **Birgit Sehested Hansen:** Conceptualization; Data curation; Formal analysis. **William A Hagopian:** Conceptualization; Data curation; Formal analysis; Funding acquisition; Investigation; Methodology; Project administration; Resources; Software; Supervision; Validation; Visualization; Writing‐original draft; Writing‐review & editing. **Matthias G von Herrath:** Conceptualization; Data curation; Formal analysis; Funding acquisition; Investigation; Methodology; Project administration; Resources; Software; Supervision; Validation; Visualization; Writing‐original draft; Writing‐review & editing.

## Data Availability

Data are available on request to the authors.
