# Supplementary material for: Peripheral autoreactive CD8 T‐cell frequencies are too variable to be a reliable predictor of disease progression of human type 1 diabetes
Source: Clin Transl Immunology. 2021 Jul 11;10(7):e1309. doi: 10.1002/cti2.1309 (PMC8273427; doi:10.1002/cti2.1309)

Supplementary table 1.

Participant Genotyping information. **Top:** Detailed HLA DR-DQ haplogenotypes on the 40 analyzed participants, and what percent of those in each HLA group progressed to clinical T1D during the study period. **Middle:** Distribution of HLA-A *02 genotypes among analyzed participants. **Bottom:** Distribution of Insulin Promoter VNTR class among analyzed participants.

Genotype Category	Total	T1D*	NP**
**HLA DR-DQ**			
DR3-DQ2.5/DR4-DQ8.1	12 (30%)	2 (33%)	10 (29%)
DR4-DQ8.1/DR4-DQ8.1	10 (25%)	1 (17%)	9 (26%)
DR3-DQ2.5/DR3-DQ2.5	2 (5%)	0 (0%)	2 (6%)
DR4-DQ8.1/X	7 (18%)	2 (33%)	5 (15%)
DR3-DQ2.5/ X	9 (23%)	1 (17%)	8 (24%)
**HLA-A**			
02:01:01/ 02:01:01	2 (5%)	1 (17%)	1 (3%)
02:01:01/Y	20 (50%)	1 (17%)	19 (56%)
no 02:01:01	18 (45%)	4 (67%)	14 (41%)
**INS promoter VNTR**			
I/I	21 (52%)	4 (67%)	17 (50%)
I/III	18 (45%)	2 (33%)	16 (47%)
III/III	1 (3%)	0 (0%)	1 (3%)

* T1D = developed T1D during the study.

** NP= did not progress to T1D during the study but remain at risk

X ≠ DR3-DQ2.5 or DR4-DQ8.1; Y ≠ 02:01:01

Supplementary table 2

Multimer^{+} detection per visit for all subjects positive for any autoreactive CD8 subset evaluated.
All HLA-A2:01^{+} subjects who had at least one autoantigen-specific T-cell population detectable on at least one visit are included in the table. The specificity of the T-cell population is indicated for each visit.

Subject	v1	v2	v3	v4	v5	v6	v7	v8	v9	v10	v11	v12	v13
P10				IGRP	IGRP	IGRP		IGRP					
P12								IA2; GAD65					
P14	IGRP	IGRP	IGRP	IGRP	IGRP			IGRP	IGRP	IGRP	IGRP	IGRP	IGRP
P17													IA2; GAD65; PPI
P18		PPI					PPI						
P22					PPI	IA2		IA2					
P26	IA2; InsB				IA2; InsB; PPI	IA2; InsB; PPI	InsB; ppIAPP; PPI	InsB; ppIAPP; PPI	IA2;InsB ; ppIAPP; PPI	IA2; InsB; PPI		IA2;InsB; ppIAPP; PPI	InsB; PPI
P38										PPI			
P49					IA2								
P50			IA2; IGRP; InsB; PPI; ppIAPP	PPI; InsB		IA2; PPI; InsB		InsB		IA2; PPI			
P54													GAD65
P56						InsB	InsB						

Supplementary table 3

Application summary for the flow cytometry panel detailing antibody clones, vendors, LSRII laser and filters used to phenotype circulating NK cells and monocyte subsets (NK/Mono panel).

**Purpose**	Phenotype basic monocyte and NK cell subsets ex vivo			
**Species**	Human PBMC			
**Cell type(s)**	Monocytes & NK cells			
**Cross Reference**	None			
**Instrument**	LSR II	**HTS or Tube?**	Either	
**Anti-body (Clone)**	**Fluorochrome**	**Vendor**	**Laser**	**Filters**
**Anti-CD57 (HCD57)**	BV421 (Pacific Blue)	Biolegend, San Diego, CA, USA	Violet	450/50 (no LP)
**Anti-CD36 (CB38)**	BV605	BD Bioscience, San Diego, CA, USA	Violet	610/20 (595 LP)
**Anti-CD14 (M5E2)**	BV711	Biolegend	Violet	710/40 (685 LP)
**Anti-HLADR,DQ,DP (Tu39)**	FITC	BD	Blue	525/50 (505 LP)
**Anti-CD206 (1-52)**	PerCPCy5.5	Biolegend	Blue	695/40 (635 LP)
**Anti-NKp46 (9E2/NKp46)**	PE	BD	YellowGreen	582/15 (no LP)
**Anti-CD3 (UCHT1)**	ECD (PETxRD)	Beckman coulter, Brea, CA, USA	YellowGreen	610/20 (600 LP)
**Anti-CD19 (J4-119)**	ECD (PETxRD)	Beckman coulter	YellowGreen	610/20 (600 LP)
**Anti-CD54 (HA58)**	PECy5	BD	YellowGreen	670/30 (635 LP)
**Anti-CD56 (NCAM16.2)**	PECy7	BD	YellowGreen	780/60 (750 LP)
**Anti-NKG2D (1D11)**	APC	BD	Red	670/30 (no LP)
**Anti-CD16 (3G8)**	APCH7 (APCCy7)	BD	Red	780/60 (750 LP)
**Anti-CD2 (RPA2.10)**	BUV395	BD	UV	379/28 (no LP)
**Anti-CD274 (MIH1)**	BV786 (Qdot 800)	BD	Violet	800/30 (750 LP)
**LDA**	AmCyan	Life Technologies, Carlsbad, CA,USA	Violet	525/50 (505 LP)

Supplementary table 4

NK/Monocyte flow cytometry panel parameter definitions

Population	***Definition***	***Details***
**Live Cells**	Singlets, Live	*Percentage of single live cells*
**NK Cells**	Singlets, Live, Dump$^-$, size, CD14$^-$, CD56$^+$,CD16$^+$	*Percentage of CD14$^-$ Lymphocyte events*
**NK Cells CD2$^+$**	Singlets, Live, Dump$^-$, size, CD14$^-$, CD56$^+$,CD16$^+$ CD2$^+$	*Percentage of NK cells*
**NK Cells CD36$^+$**	Singlets, Live, Dump$^-$, size, CD14$^-$, CD56$^+$,CD16$^+$, CD36+	*Percentage of NK cells*
**NK Cells CD54$^+$**	Singlets, Live, Dump$^-$, size, CD14$^-$, CD56$^+$,CD16$^+$, CD54$^+$	*Percentage of NK cells*
**NK Cells CD57$^+$**	Singlets, Live, Dump$^-$, size, CD14$^-$, CD56$^+$,CD16$^+$, CD57$^+$	*Percentage of NK cells*
**NK Cells NKG2D$^+$**	Singlets, Live, Dump$^-$, size, CD14$^-$, CD56$^+$,CD16$^+$, NKG2D$^+$	*Percentage of NK cells*
**NK Cells NKp46$^+$**	Singlets, Live, Dump$^-$, size, CD14$^-$, CD56$^+$,CD16$^+$, NKp46$^+$	*Percentage of NK cells*
**NK^{high} Cells**	Singlets, Live, Dump$^-$, size, CD14$^-$, CD56^{hi},CD16$^-$	*Percentage of CD14$^-$ Lymphocyte events*
**NK^{high} Cells CD2$^+$**	Singlets, Live, Dump$^-$, size, CD14$^-$, CD56^{hi},CD16$^-$, CD2$^+$	*Percentage of NK^{high} cells*
**NK^{high} Cells CD36$^+$**	Singlets, Live, Dump$^-$, size, CD14$^-$, CD56^{hi},CD16$^-$, CD36$^+$	*Percentage of NK^{high} cells*
**NK^{high} Cells CD54$^+$**	Singlets, Live, Dump$^-$, size, CD14$^-$, CD56^{hi},CD16$^-$, CD54$^+$	*Percentage of NK^{high} cells*
**NK^{high} Cells CD57$^+$**	Singlets, Live, Dump$^-$, size, CD14$^-$, CD56^{hi},CD16$^-$, CD57$^+$	*Percentage of NK^{high} cells*
**NK^{high} Cells NKG2D$^+$**	Singlets, Live, Dump$^-$, size, CD14$^-$, CD56^{hi},CD16$^-$, NKG2D$^+$	*Percentage of NK^{high} cells*
**NK^{high} Cells NKp46$^+$**	Singlets, Live, Dump$^-$, size, CD14$^-$, CD56^{hi},CD16$^-$, NKp46$^+$	*Percentage of NK^{high} cells*
**CD14^{high} Monocytes**	Singlets, Live, Dump$^-$, size, CD56$^-$, CD14^{hi}	*Percentage of CD56$^-$ Monocyte events*
**CD14^{high} Monocytes CD2$^+$**	Singlets, Live, Dump$^-$, size, CD56$^-$, CD14^{hi}, CD2$^+$	*Percentage of CD14^{high} Monocyte events*
**CD14^{high} Monocytes CD36$^+$**	Singlets, Live, Dump$^-$, size, CD56$^-$, CD14^{hi}, CD36$^+$	*Percentage of CD14^{high} Monocyte events*
**CD14^{high} Monocytes CD57$^+$**	Singlets, Live, Dump$^-$, size, CD56$^-$, CD14^{hi}, CD57$^+$	*Percentage of CD14^{high} Monocyte events*
**CD14^{high} Monocytes HLA CLASS II$^+$**	Singlets, Live, Dump$^-$, size, CD56$^-$, CD14^{hi}, HLA CLASS II$^+$	*Percentage of CD14^{high} Monocyte events*
**CD14^{high} Monocytes PDL1$^+$**	Singlets, Live, Dump$^-$, size, CD56$^-$, CD14^{hi}, PDL1$^+$	*Percentage of CD14^{high} Monocyte events*
**CD14^{low} monocytes**	Singlets, Live, Dump$^-$, size, CD56$^-$, CD14^{lo}	*Percentage of CD56$^-$ Monocyte events*
**CD14^{low} Monocytes CD2$^+$**	Singlets, Live, Dump$^-$, size, CD56$^-$, CD14^{lo}, CD2$^+$	*Percentage of CD14^{low} Monocyte events*
**CD14^{low} monocytes CD36$^+$**	Singlets, Live, Dump$^-$, size, CD56$^-$, CD14^{lo}, CD36$^+$	*Percentage of CD14^{low} Monocyte events*
**CD14^{low} monocytes CD57$^+$**	Singlets, Live, Dump$^-$, size, CD56$^-$, CD14^{lo}, CD57$^+$	*Percentage of CD14^{low} Monocyte events*
**CD14^{low} monocytes HLA CLASS II$^+$**	Singlets, Live, Dump$^-$, size, CD56$^-$, CD14^{lo}, HLA CLASS II$^+$	*Percentage of CD14^{low} Monocyte events*
**CD14^{low} monocytes PDL1$^+$**	Singlets, Live, Dump$^-$, size, CD56$^-$, CD14^{lo}, PDL1$^+$	*Percentage of CD14^{low} Monocyte events*

Supplementary table 5

Antigen-fluorochrome combinations used for detection of antigen-specific CD8$^+$ T-cells.

pHLA Monomer	Peptide Sequence	SAQdot Multimer #1	SAQdot Multimer #2
**CMV pp65**	NLVPMVATV	Qdot 585	Qdot 800
**EBV LMP2**	CLGGLLTMV	Qdot 585	Qdot 800
**Measles H250**	SMYRVFEVGV	Qdot 585	Qdot 800
**IGRP**	VLFGLGFAI	Qdot 585	Qdot 655
**InsB[10-18]**	HLVEALYLV	Qdot 605	Qdot 655
**ppIAPP**	KLQVFLIVL	Qdot 605	Qdot 800
**PPI**	ALWGPDPAAA	Qdot 655	Qdot 705
**GAD65**	VMNILLQYVV	Qdot 585	Qdot 705
**IA2**	MVWESGCTV	Qdot 585	Qdot 605

CMV pp65*, cytomegalovirus phosphoprotein 65;* ***EBV LMP2****, Epstein-Barr virus latent membrane protein 2;* ***H250****, hemagglutinin protein from measles virus;* ***GAD65****, glutamic acid decarboxylase 65;* ***IGRP****, islet-specific glucose-6-phosphatase catalytic subunit-related protein;* ***IA2****, islet tyrosine phosphatase 2 (insulinoma antigen-2);* ***InsB****, insulin B;* ***pHLA****, peptide-human leukocyte antigen complex;* ***ppIAPP****, prepro islet amyloid polypeptide;* ***PPI****, pre-proinsulin;* ***SAQdot****, streptavidin-conjugated quantum dot*

Supplementary table 6

Application summary for the flow cytometry panel used to evaluate and phenotype circulating T-cell subsets (QDM panel).

**Purpose**	Determine the frequency of diabetogenic effector, effector memory, & TEMRA CD8 Tcells			
**Species**	Human			
**Cell type(s)**	PBMC (cryopreserved)			
**Cross Reference**	Hadrup et al. 2009, Velthuis et al. 2010, Andersen et al. 2012			
**Instrument**	LSRII	**HTS or Tube?**	Tube	
**Anti-body (Clone)**	**Fluorochrome**	**Vendor**	**Laser**	**Filters**
**Anti-CD8 (RPAT8)**	APC	BD Pharmigen	Red	670/30 No LP
**Anti-CD4 (SKe)**	APCH7	BD Biosciences	Red	780/60 750 LP
**Anti-CD14 (HCD14)**	FITC	BD Biosciences	Blue	525/50 505 LP
**Anti-CD16 (3G8)**	FITC	BD Pharmigen	Blue	525/50 505 LP
**Anti-CD20 (2H7)**	FITC	BD Biosciences	Blue	525/50 505 LP
**Anti-CD40 (5C3)**	FITC	BD Pharmigen	Blue	525/50 505 LP
**Anti-CD56 (NCAM16.2)**	FITC	BD Biosciences	Blue	525/50 505 LP
**Anti-CD45RA (H1100)**	Alexa Fluor 700	BD Biosciences	Red	720/40 685 LP
**Anti-CD197 (150503)**	BV450	BD Biosciences	Violet	450/50 No LP
**Anti-CD183 (IC6)**	PE	BD Pharmigen	Yellow/ Green	582/15 555 LP
**LDA**	AmCyan	Life Technologies	Violet	525/50 505 LP

Supplementary table 7

QDM flow cytometry panel parameter definitions.

CM, central memory; EM, effector memory; N, naïve; TEMRA, terminally differentiated CD45RA$^+$

Population	Definition	Details
Live Cells	Singlet,/Live_Dead-	Percentage of single events
CD4$^+$	Singlet/Live_Dead-/Dump-/CD4$^+$	Percentage of Dump- events
CD4$^+$ CM	Singlet/Live_Dead-/Dump-/CD4$^+$/CD45RA$^-$/CCR7$^+$	Percentage of Total CD4$^+$ T-cells
CD4$^+$ CXCR3$^+$	Singlet/Live_Dead-/Dump-/CD4$^+$/CXCR3$^+$	Percentage of Total CD4$^+$ T-cells
CD4$^+$ EM	Singlet/Live_Dead-/Dump-/CD4$^+$/CD45RA$^-$/CCR7$^-$	Percentage of Total CD4$^+$ T-cells
CD4$^+$ N	Singlet/Live_Dead-/Dump-/CD4$^+$/CD45RA$^+$/CCR7$^+$	Percentage of Total CD4$^+$ T-cells
CD4$^+$ TEMRA	Singlet/Live_Dead-/Dump-/CD4$^+$/CD45RA$^+$/CCR7$^-$	Percentage of Total CD4$^+$ T-cells
CD8$^+$	Singlet/Live_Dead-/Dump-/CD8$^+$	Percentage of Dump- events
CD8$^+$ CM	Singlet/Live_Dead-/Dump-/CD8$^+$/CD45RA$^-$/CCR7$^+$	Percentage of Total CD8$^+$ T-cells
CD8$^+$ CXCR3$^+$	Singlet/Live_Dead-/Dump-/CD8$^+$/CXCR3$^+$	Percentage of Total CD8$^+$ T-cells
CD8$^+$ EM	Singlet/Live_Dead-/Dump-/CD8$^+$/CD45RA$^-$/CCR7$^-$	Percentage of Total CD8$^+$ T-cells
CD8$^+$ N	Singlet/Live_Dead-/Dump-/CD8$^+$/CD45RA$^+$/CCR7$^+$	Percentage of Total CD8$^+$ T-cells
CD8$^+$ TEMRA	Singlet/Live_Dead-/Dump-/CD8$^+$/CD45RA$^+$/CCR7$^-$	Percentage of Total CD8$^+$ T-cells
Total CD8$^+$ IA2$^+$	Singlet/Live_Dead-/Dump-/CD8$^+$/CD8 N$^-$/Qdot655$^-$ /Qdot705$^-$/Qdot800$^-$/Qdot585$^+$/ Qdot605$^+$	Percentage of Total CD8$^+$ T-cells
Total CD8$^+$ PPI$^+$	Singlet/Live_Dead-/Dump-/CD8$^+$/CD8 N-/Qdot585$^-$ /Qdot605$^-$/Qdot800$^-$/Qdot655$^+$/ Qdot705$^+$	Percentage of Total CD8$^+$ T-cells
Total CD8$^+$ ppiAPP$^+$	Singlet/Live_Dead-/Dump-/CD8$^+$/CD8 N$^-$/Qdot585$^-$ /Qdot655$^-$/Qdot705$^-$/Qdot605$^+$/ Qdot800$^+$	Percentage of Total CD8$^+$ T-cells
Total CD8$^+$ InsB$^+$	Singlet/Live_Dead-/Dump-/CD8$^+$/CD8 N$^-$/Qdot585$^-$ /Qdot705$^-$/Qdot800$^-$/Qdot605$^+$/ Qdot655$^+$	Percentage of Total CD8$^+$ T-cells
Total CD8$^+$ IGRP$^+$	Singlet/Live_Dead-/Dump-/CD8$^+$/CD8 N$^-$/Qdot605$^-$ /Qdot705$^-$/Qdot800$^-$/Qdot585$^+$/ Qdot655$^+$	Percentage of Total CD8$^+$ T-cells
Total CD8$^+$ VIRAL$^+$	Singlet/Live_Dead-/Dump-/CD8$^+$/CD8 N$^-$/Qdot605$^-$ /Qdot655$^-$/Qdot705$^-$/Qdot585$^+$/ Qdot800$^+$	Percentage of Total CD8$^+$ T-cells
Total CD8$^+$ GAD65$^+$	Singlet/Live_Dead-/Dump-/CD8$^+$/CD8 N$^-$/Qdot605$^-$ /Qdot655-/Qdot800$^-$/Qdot585$^+$/ Qdot705$^+$	Percentage of Total CD8$^+$ T-cells

Supplementary figure 1. %HbA1c overtime for all subjects. The perpendicular line indicates diagnosis of T1D.

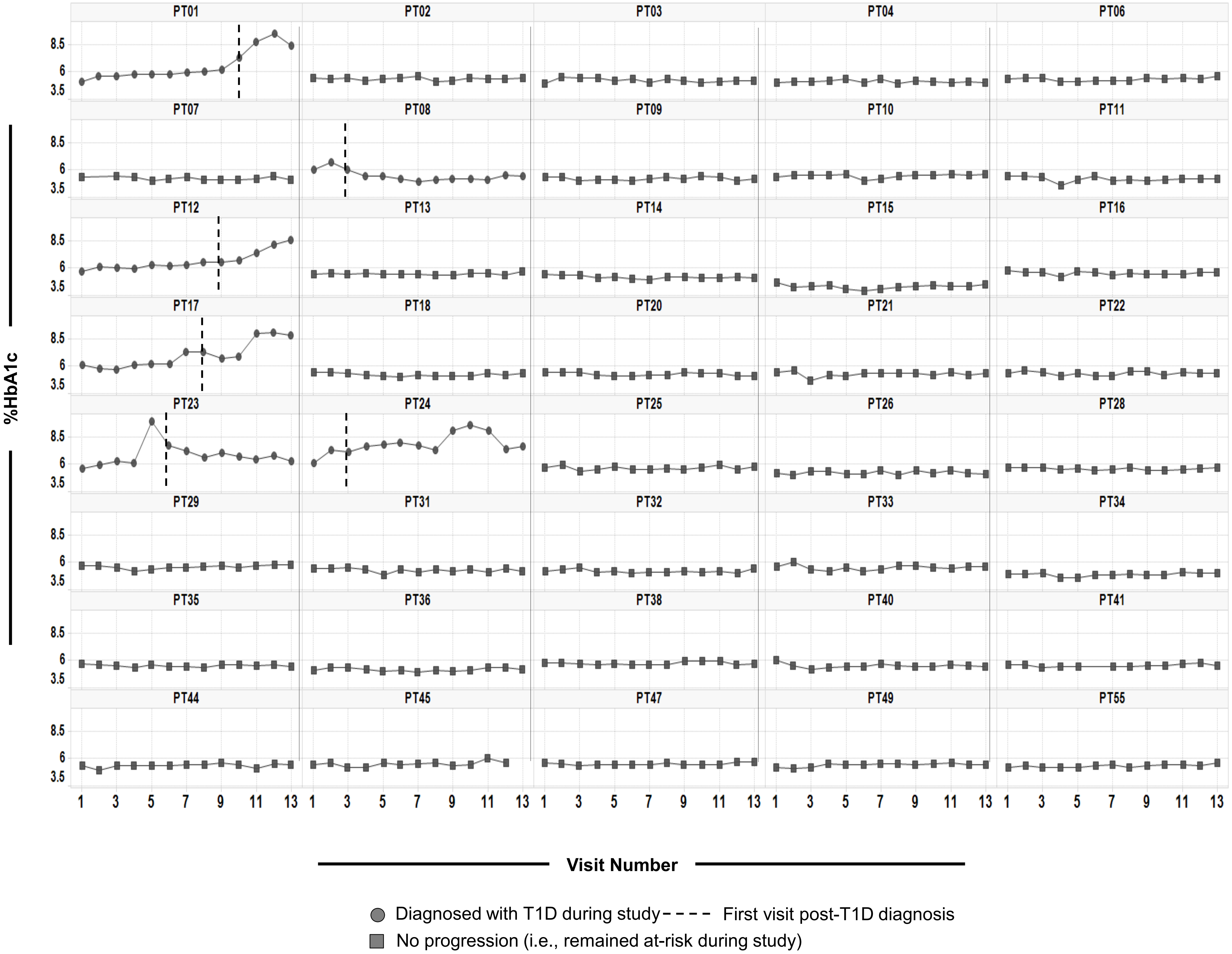

Supplementary figure 2. $CD4^+$ T-cell populations are shown for each of the 6 study participants who were diagnosed with type 1 diabetes during the study period. The dotted line indicates the visit following diagnosis.

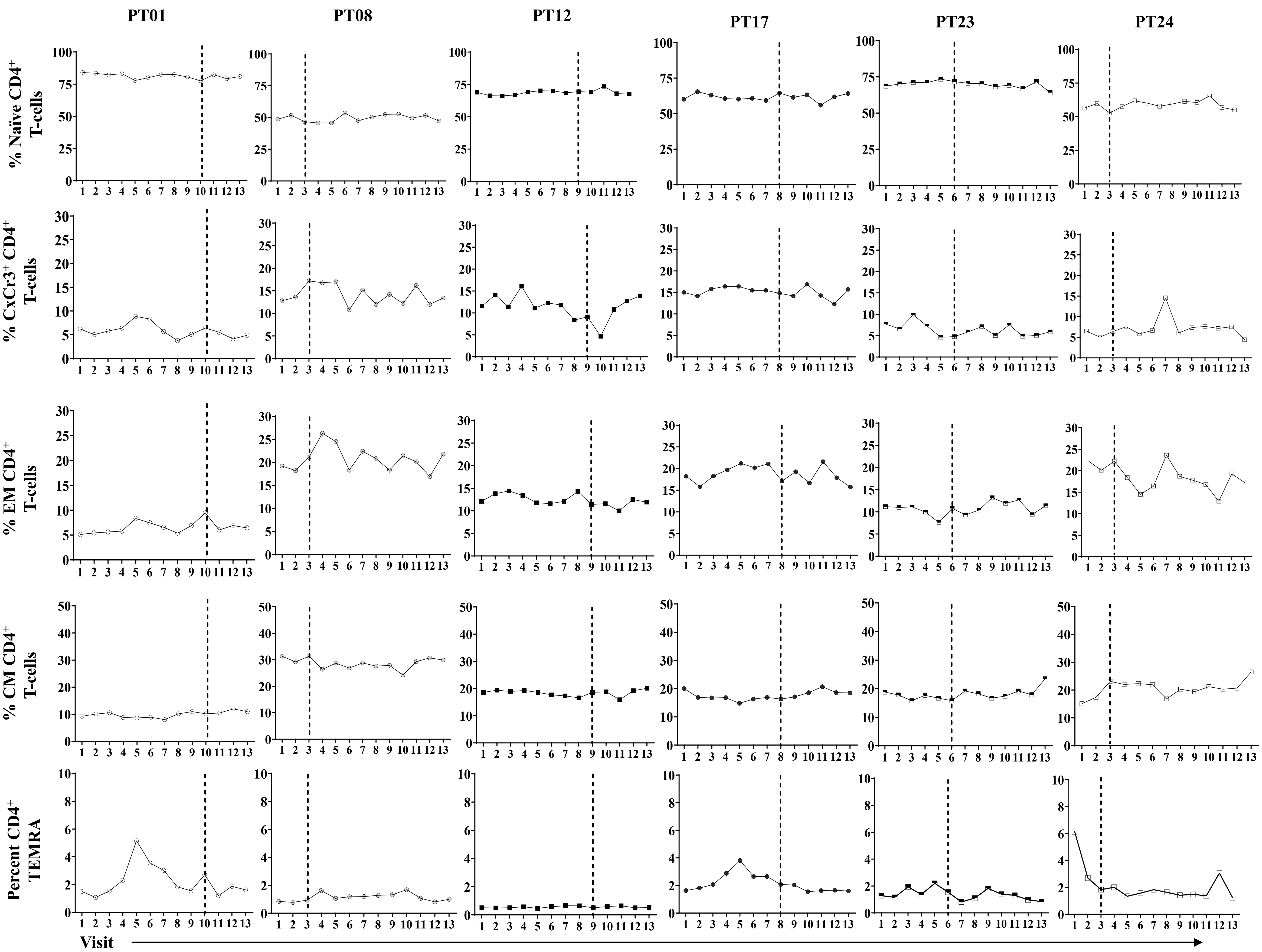

Supplementary figure 3. CD8⁺ T-cell populations are shown for each of the 6 study participants who were diagnosed with type 1 diabetes during the study period. The dotted line indicates the visit following diagnosis.

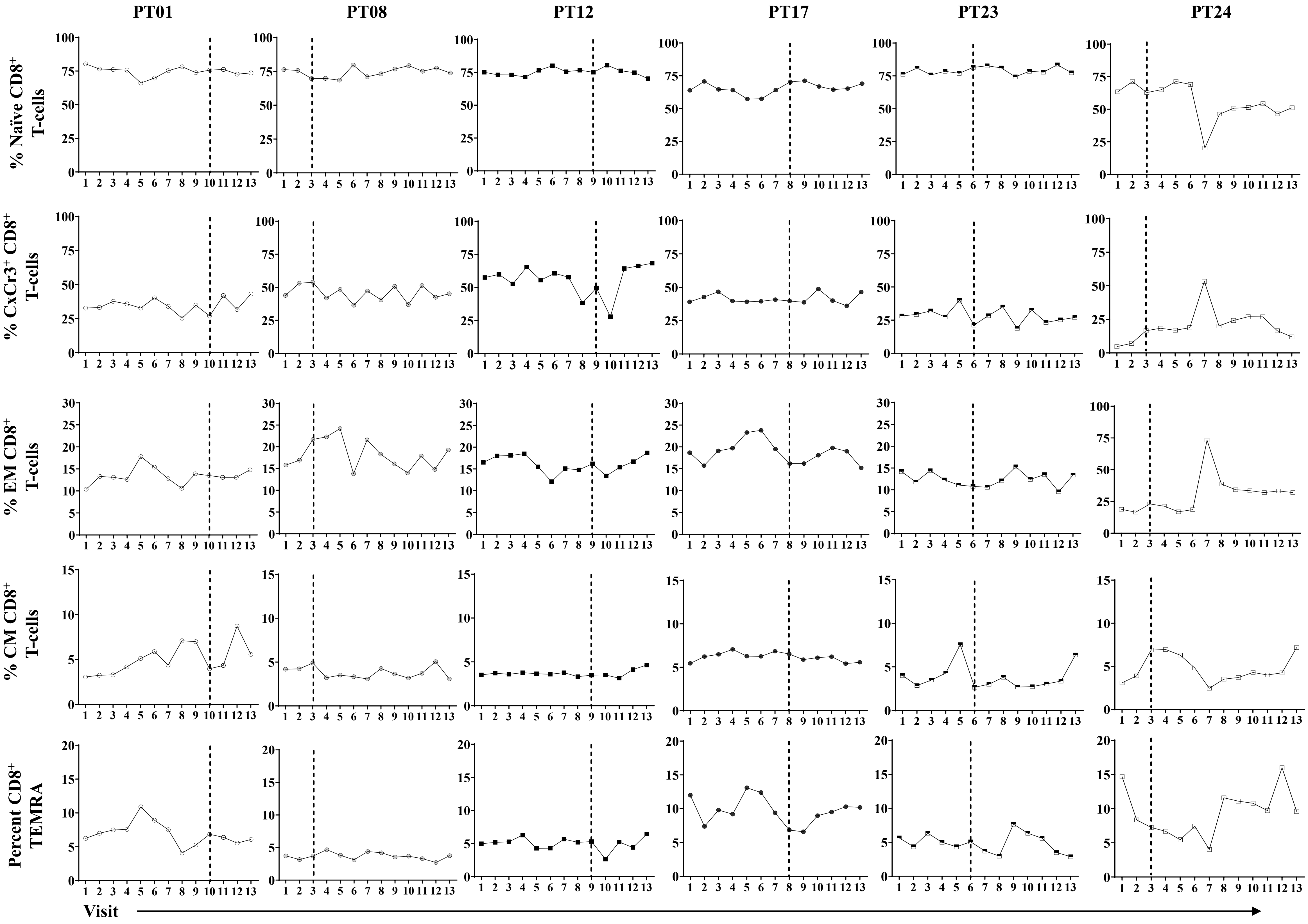

Supplementary figure 4. NK and monocyte populations are shown for each of the 6 study participants who were diagnosed with type 1 diabetes during the study period. The dotted line indicates the visit following diagnosis.

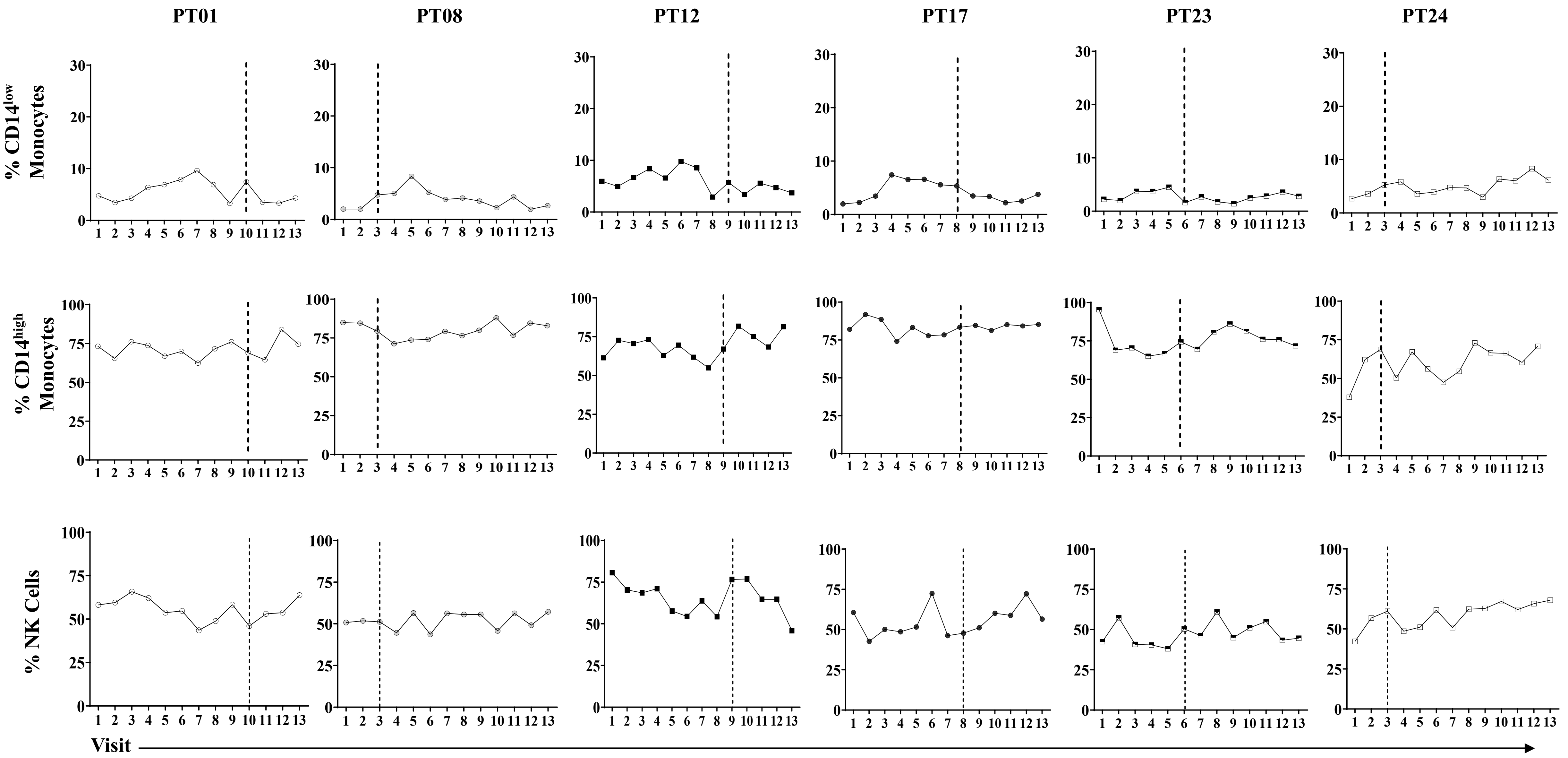

Supplementary figure 5. There are no significant differences in the frequency of autoreactive T-cells regardless of disease status.

Combined frequencies off all autoreactive CD8+ T-cell populations are show per subject. **(a)** The total autoreactive $CD8^+$ T-cell populations are show for each HLA-A2:01$^+$ individual. **(b)** Shows $CxCr3^+$ autoreactive $CD8^+$ T-cells. The grey and black dotted-line arrows indicate the date of diagnosis for individuals represented by the the grey and black circle, respectively.

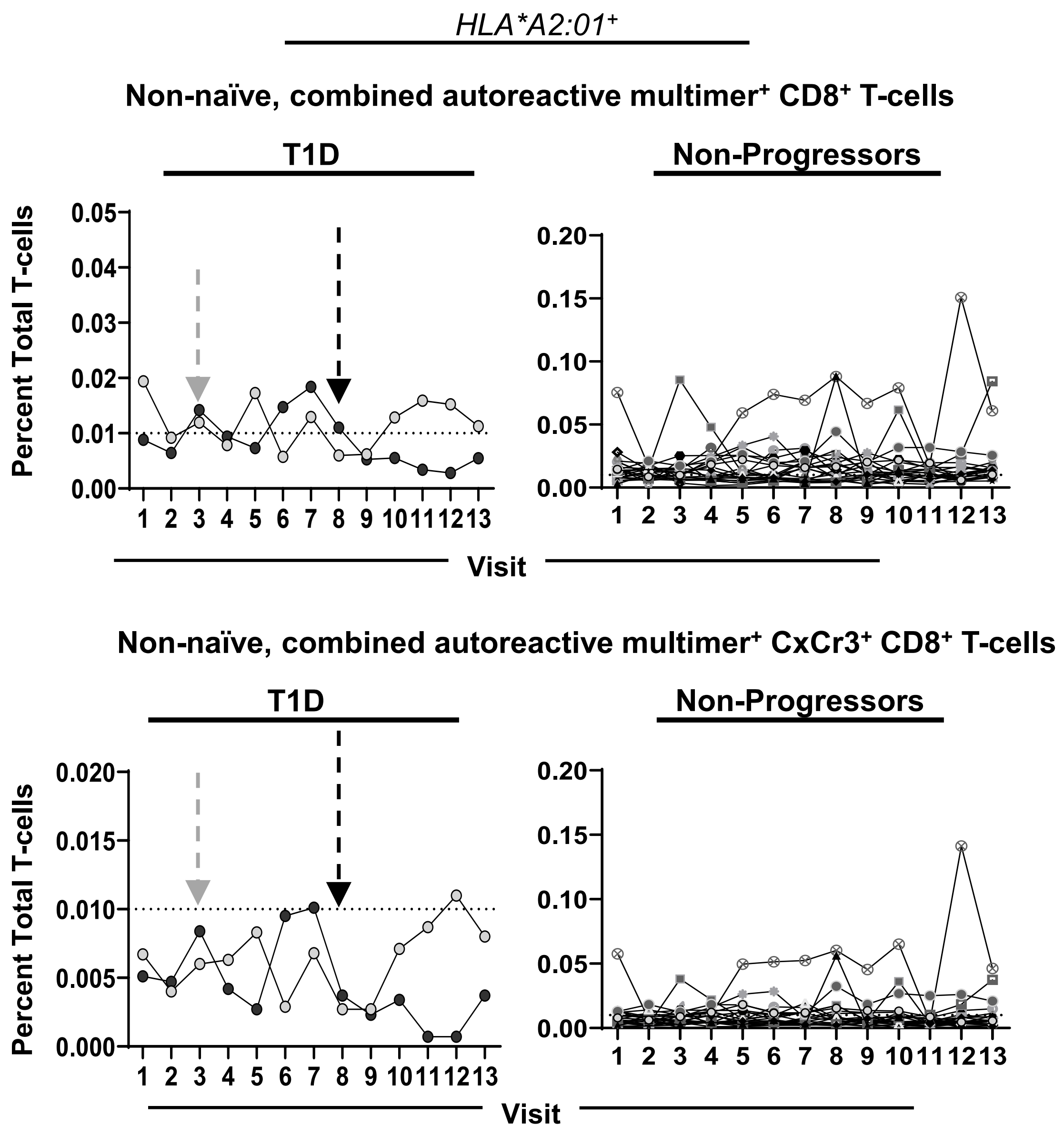

Supplementary figure 6. There are no differences in autoreactive frequency between subjects who were diagnosed with T1D during the study versus those who did not progress.

All positive autoreactive multimer (anti-GAD65, -InsB, -IGRP, -ppiAPP, -PPI) events per visit are combined to provide a composite frequency. The mean percentage is shown for each group, and the error bars represent the standard deviation of the mean. **(a)** The overall autoreactive CD8+ T-cell frequency is show for the HLA-A2:01+ subjects who were diagnosed with T1D during the study (●) compared to the HLA-A2:01+ subjects who remained non-diabetic throughout the study (■). **(b)** The frequency of CxCr3+ autoreactive CD8+ T-cells is shown for the HLA-A2:01+ T1D vs. non-T1D.

(a)

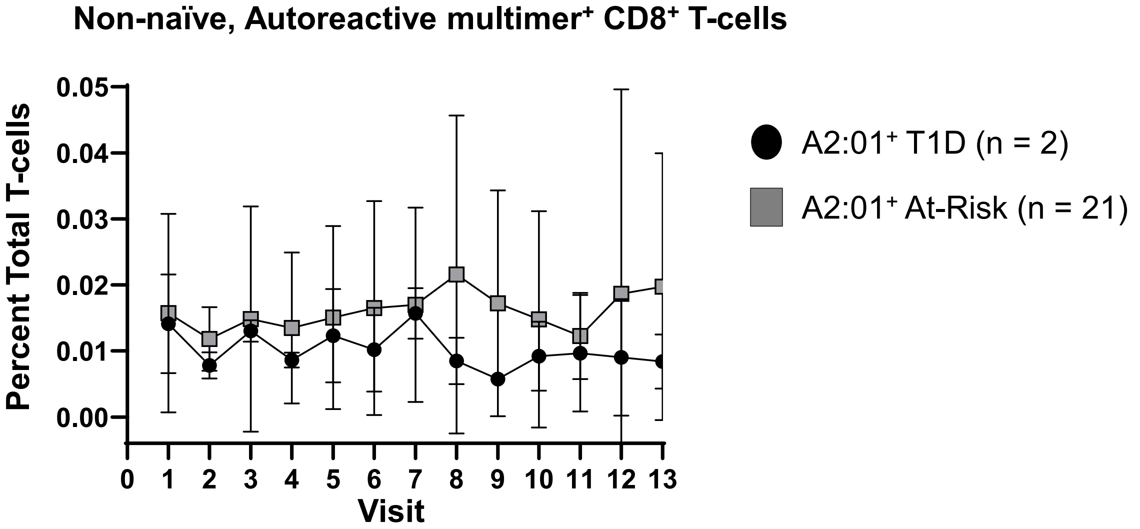

(b)

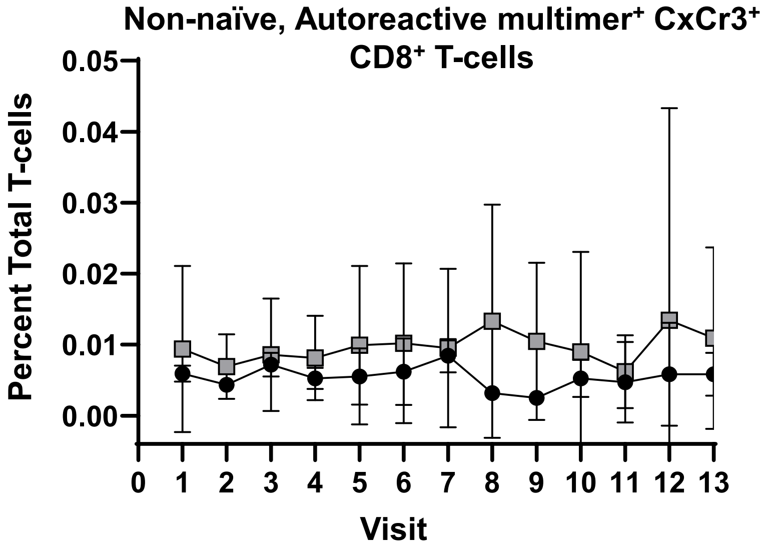

Supplementary figure 7. There are no differences in autoreactive frequency between subjects who were single or multiple autoantibody-positive compared to those diagnosed with T1D during the study.

All positive autoreactive multimer (anti-GAD65, -InsB, -IGRP, -ppiAPP, -PPI) events per visit are combined to provide a composite frequency. The non-progressing HLA-A2:01^{+} subjects were then split into single or >2 AAbs at enrolment. HLA-A2:01^{+} subjects diagnosed with T1D during the study (n = 2) The mean percentage is shown for each group, and the error bars represent the standard deviation of the mean. **(a)** The overall autoreactive CD8+ T-cell frequency is shown for the HLA-A2:01^{+} subjects who were single AAb^{+} at enrolment (◯) vs. HLA-A2:01^{+} subjects who were positive for >2 AAbs (◇) vs. T1D (■). **(b)** The frequency of CxCr3^{+} autoreactive CD8^{+} T-cells is shown for the HLA-A2:01^{+} who were either single AAb^{+} or multiple AAb^{+} at enrolment, or who were diagnosed with T1D during study.

(a)

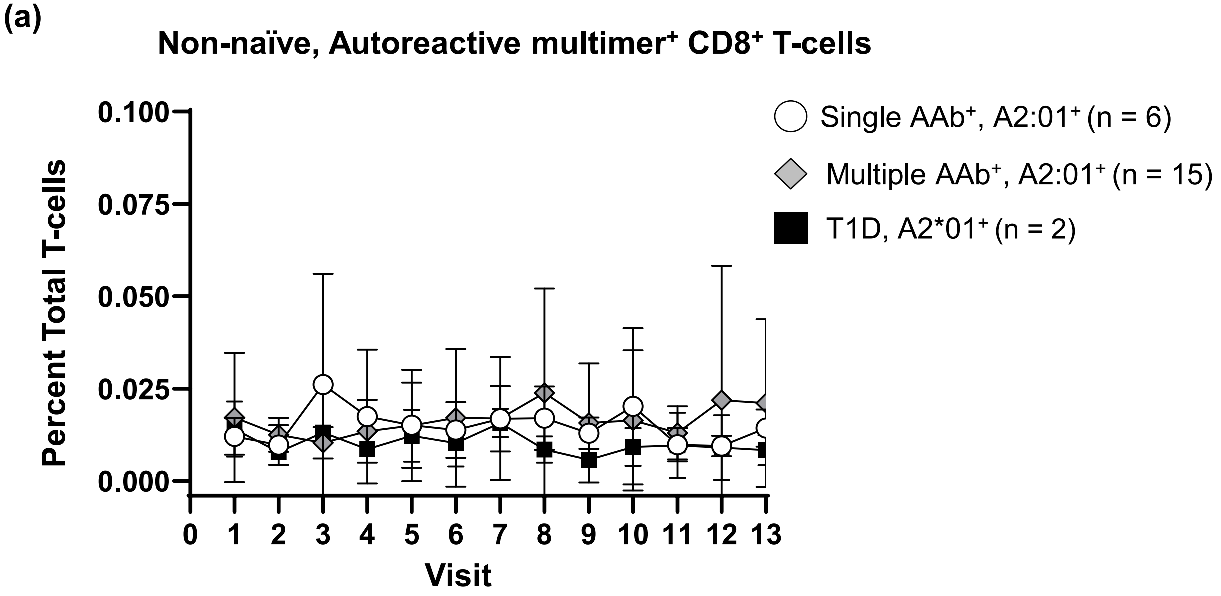

(b)

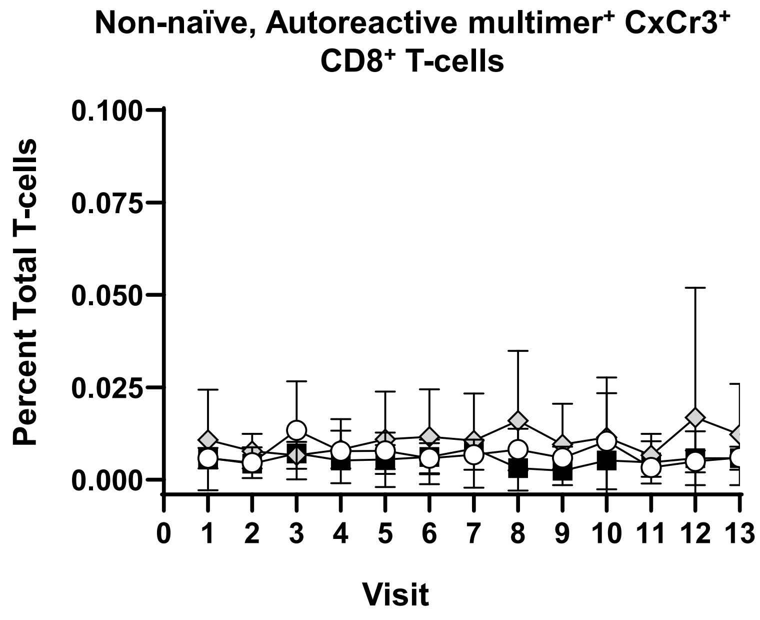

Supplementary figure 8.
Representative data and gating for the NK/Mono flow cytometry panel. Representative monocyte **(a)** and NK cell **(b)** subset analyses.

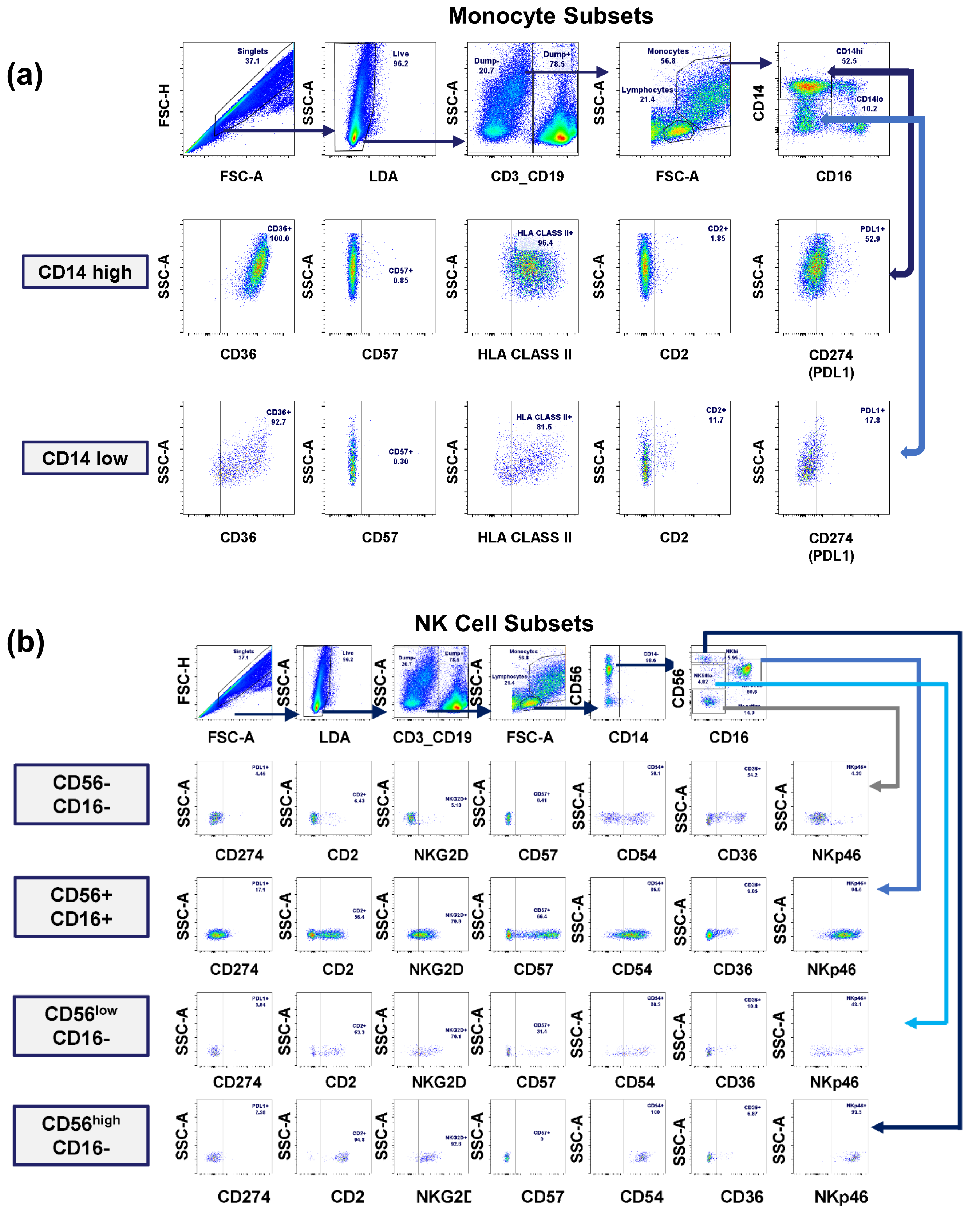

Supplementary figure 9.

Gating scheme used for the analyses of T-cell subsets, including multimer⁺ cells. **(a)** Illustration of the quantum dot background exclusion (QBE) gating strategy used. This is a basic gating strategy to exclude potential background signal introduced by Qdots to each multimer pair**. (b)** Gating strategy for identifying T-cell subsets.

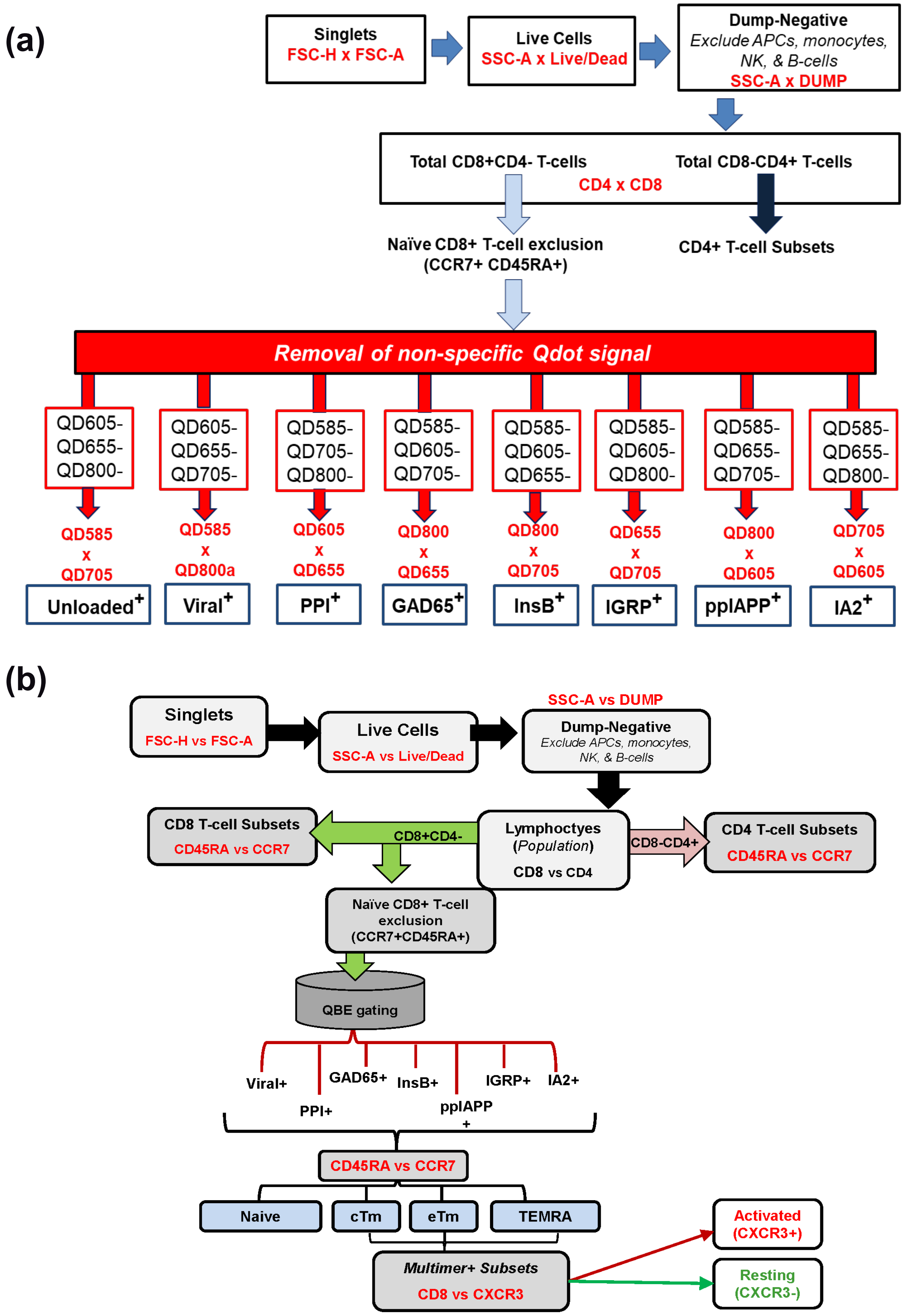

Supplementary figure 10.
Representative data for T-cell subset analyses. **(a)** Representative data showing gating scheme of major T cell populations and exclusion of Qdot fluorescence. The dotted, red lines highlight populations that are excluded from further analyses. **(b)** Representative multimer analyses showing a PPI⁺ and CMV⁺ control sample.

(a)

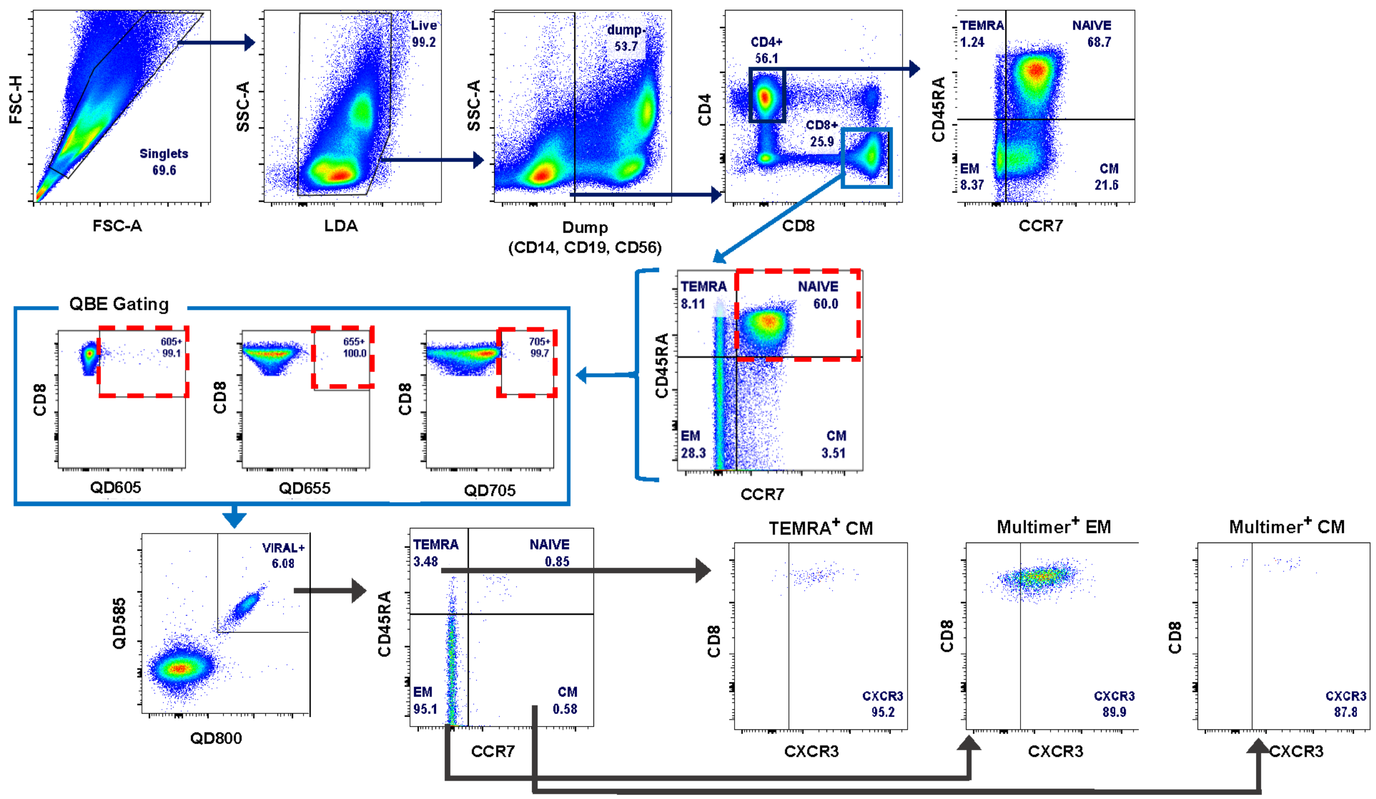

(b)

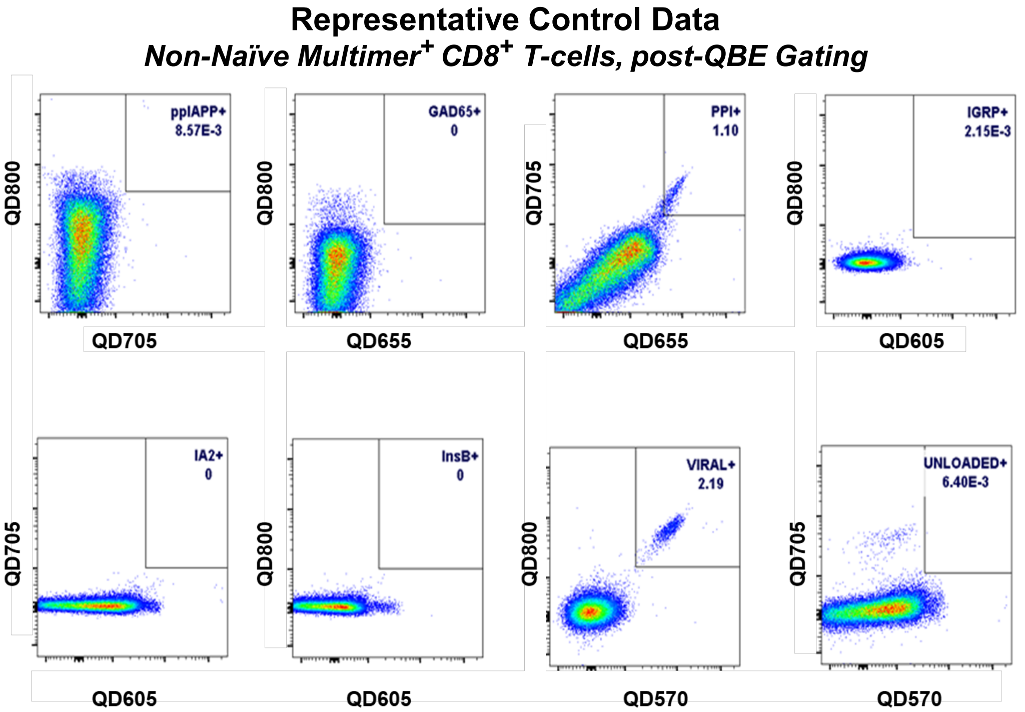

Supplement: Supplementary file 1 [file CTI2-10-e1309-s001.pdf]
